# The RNA-binding protein hnRNP F is required for the germinal center B cell response

**DOI:** 10.1038/s41467-023-37308-z

**Published:** 2023-03-30

**Authors:** Hengjun Huang, Yuxing Li, Gaopu Zhang, Gui-Xin Ruan, Zhijian Zhu, Wenjing Chen, Jia Zou, Rui Zhang, Jing Wang, Yu Ouyang, Shengli Xu, Xijun Ou

**Affiliations:** 1 School of Life Sciences, Southern University of Science and Technology, Shenzhen, 518055 China; 2 Medical School, Taizhou University, Taizhou, 318000 China; 3 Department of Computer Science and Engineering, College of Engineering, Southern University of Science and Technology, Shenzhen, 518055 China; 4 Singapore Immunology Network, Agency for Science, Technology and Research, Singapore, 138648 Singapore; 5 Department of Physiology, Yong Loo Lin School of Medicine, National University of Singapore, Singapore, 117599 Singapore

**Keywords:** B cells, Germinal centres, Gene regulation in immune cells, RNA splicing

## Abstract

The T cell-dependent (TD) antibody response involves the generation of high affinity, immunoglobulin heavy chain class-switched antibodies that are generated through germinal center (GC) response. This process is controlled by coordinated transcriptional and post-transcriptional gene regulatory mechanisms. RNA-binding proteins (RBPs) have emerged as critical players in post-transcriptional gene regulation. Here we demonstrate that B cell-specific deletion of RBP hnRNP F leads to diminished production of class-switched antibodies with high affinities in response to a TD antigen challenge. B cells deficient in hnRNP F are characterized by defective proliferation and c-Myc upregulation upon antigenic stimulation. Mechanistically, hnRNP F directly binds to the G-tracts of *Cd40* pre-mRNA to promote the inclusion of *Cd40* exon 6 that encodes its transmembrane domain, thus enabling appropriate CD40 cell surface expression. Furthermore, we find that hnRNP A1 and A2B1 can bind to the same region of *Cd40* pre-mRNA but suppress exon 6 inclusion, suggesting that these hnRNPs and hnRNP F might antagonize each-other’s effects on *Cd40* splicing. In summary, our study uncovers an important posttranscriptional mechanism regulating the GC response.

## Abstract (web summary)

The germinal centre (GC) response is characterized by regulated production of high affinity, class-switched antibodies in response to T-cell dependent antigens. Here authors show that the GC response is not only regulated at the transcriptional and protein levels, but also by the RNA-binding protein hnRNP F via alternative splicing of the co-stimulatory molecule CD40.

## Introduction

RNA-binding proteins (RBP) have emerged as critical players in the post-transcriptional regulation of gene expression^1^. Heterogeneous nuclear ribonucleoproteins (hnRNP) are a large family of typical RBPs expressed abundantly in mammalian cells and comprising about 30 members, including 20 major types of hnRNPs A to U and several minor types of hnRNPs^2^. Four different types of RNA-binding domains (RBD) are found in hnRNP proteins, including RNA recognition motif (RRM), non-classical quasi-RRM (q-RRM), glycine-rich domain harboring an RGG box, and K homology (KH) domain^3^.

HnRNP F belongs to the hnRNP F/H subfamily and contains three non-classical q-RRMs^4^. The best-known function of hnRNP F/H proteins is to regulate RNA alternative splicing (AS). HnRNP F was reported to regulate AS of several important genes, such as *c-Src*, *Bcl-xl*, *Mcl-1*, and *Tcf3*, primarily via exon-related AS events^5–8^. In vitro studies have demonstrated that hnRNP F participates in AS regulation by directly binding to the G-rich pre-mRNA sequence (G-tract) through its q-RRMs^9–11^. Furthermore, early studies showed that hnRNP F could bind to the adjacent regions of the target pre-mRNA exons and recruit splicing factors U1 or U2 to promote splicing of the target exons^12,13^. In addition to regulating RNA AS, previous studies also found that hnRNP F could regulate mRNA stability, translation, and alternative polyadenylation (APA)^14–18^. It was documented that hnRNP F could modulate the APA of immunoglobulin heavy (IgH) chain mRNA, promoting the expression of membrane-anchored B cell receptors (BCR) and repressing the expression of secreted Ig proteins in mouse B cell lines^14^. However, the physiological role of hnRNP F in B cell development and activation remains unknown.

B cell development starts from the hematopoietic stem cells in the bone marrow (BM) or fetal liver^19^. Through a tightly controlled multi-step process, progenitor B cells develop into mature B cells in the BM and then populate secondary lymphoid tissues. In the periphery, naïve B cells become activated upon encountering foreign antigens and differentiate into long-lived antibody-secreting plasma cells (PC) and memory B cells, establishing lifelong humoral immunity. Most long-lived PCs and memory B cells are generated in response to T cell-dependent (TD) proteinous antigens in germinal centers (GC), specialized microstructures in the secondary lymphoid tissues, such as the spleen and lymph nodes^20^. Upon engagement by TD antigens, antigen-specific follicular B (FOB) cells upregulate chemokine receptor CCR7 that draws them towards the boundary of T and B cell zones in the spleen and lymph nodes, where they interact with CD4^+^ T cells that detect the cognate antigens presented by dendritic cells^21^. Subsequently, some activated FOB cells travel back to the center of B cell follicles and become GC B cells. GCs can be divided into the dark zone (DZ) and the light zone (LZ). In the DZ, B cells undergo robust proliferation and extensive somatic hypermutation. In contrast, in the LZ, B cells with high-affinity BCRs are positively selected through competing for the scarce antigens displayed by follicular dendritic cells and for the help of the limited number of T follicular helper cells^22^. The positively selected LZ B cells can return to DZ for further proliferation and mutation of their immunoglobulin variable region genes. After multiple rounds of shuttling, GC B cells whose BCRs are of high affinity for the specific antigens eventually differentiate into long-lived PCs and memory B cells and exit GCs^23^.

Besides the primary signal transduced by BCR, secondary signals propagated by costimulatory receptors are also essential for GC response^24^. CD40 is one of the best-characterized costimulatory molecules expressed on antigen-presenting cells, including B cells. The engagement of CD40 by CD40 ligand (CD40L) on T cells triggers multiple signaling cascades, leading to downstream immune and inflammatory responses. CD40-mediated signaling is essential for TD antibody response in almost all the stages, ranging from the interaction of cognate B and T cells, IgH chain class-switching, initiation and maturation of GCs, and antibody affinity maturation^25,26^. A recent study using CRISPR/Cas9 whole-genome screening of a human B cell line unveiled multiple previously unappreciated mechanisms for regulating the CD40 pathway^27^. Interestingly, at the post-transcriptional level, it was found that CUGBP and Elav-like family member 1 (CELF1) protein could promote *Cd40* exon 6 inclusion, leading to the expression of signaling-competent membrane-bound CD40.

Here we specifically ablate hnRNP F in B cell lineage and find that hnRNP F is required for class-switched high-affinity antibody production during TD antibody response. In the absence of hnRNP F, GC B cell formation is profoundly impaired. Furthermore, we uncover that hnRNP F is required for proper cell surface expression of CD40 in B cells by regulating the AS of *Cd40* pre-mRNA.

## Results

### B cell development is largely normal in hnRNP F-deficient mice

First, we examined if *Hnrnpf* gene was differentially expressed during B cell development by analyzing the data available in the Immunological Genome Project (ImmGen) database (https://www.immgen.org/). We found that *Hnrnpf* gene expression was comparable in all the B cell subsets, including B220^+^CD43^+^CD24^−^BP-1^−^ pre-pro-B (Hardy fraction (Fr.) A), B220^+^CD43^+^CD24^+^BP-1^−^ early pro-B (Fr. B), B220^+^CD43^+^CD24^low(lo)^BP-1^+^ late pro-B (Fr. C) and B220^+^CD43^−^IgM^+^IgD^−^ immature B (Fr. E) cells, based on the Hardy classification, in the BM, and transitional 1 (T1), T2, T3 B, marginal zone B (MZB), GC B, memory B, and plasma cells in the spleen (Supplementary Fig. 1a). We further validated the result with a real-time quantitative reverse transcription PCR (qRT-PCR) analysis to determine the *Hnrnpf* mRNA levels in the various B cell subsets sorted from wild-type mice. Consistent with the data obtained from the ImmGen database, the expression of *Hnrnpf* did not differ much amongst most of the B cell subsets, except for GC B cells that exhibited a slightly, but not statistically significant, increased expression level of *Hnrnpf* (Supplementary Fig. 1b).

**Fig. 1:**
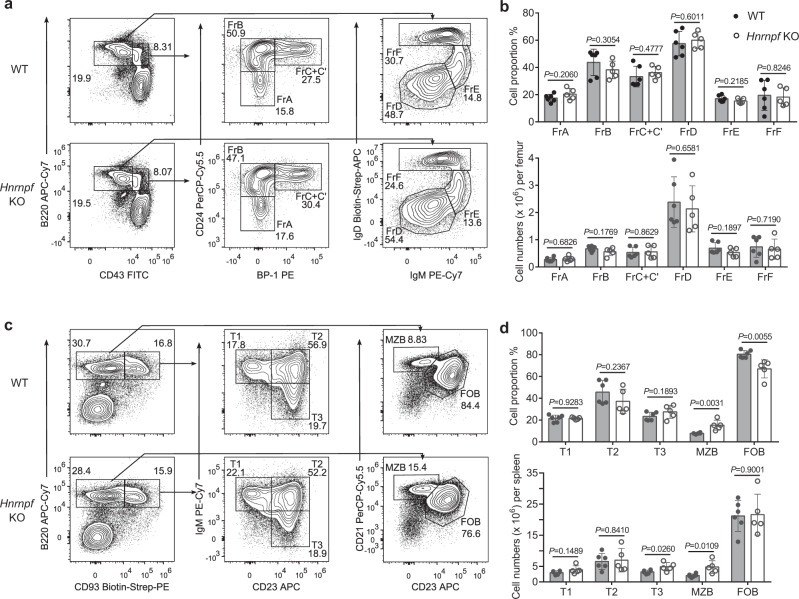
hnRNP F-deficient mice have normal B cell development. **a**, **b** Flow cytometric analysis of B cells in the BM. The frequencies (**a**) and numbers (**b**) of various B cell subsets in the BM of WT and *Hnrnpf* KO mice were analyzed and enumerated using Hardy’s gating strategy (*n* = 6 for WT group and *n* = 5 for *Hnrnpf* KO group). **c**, **d** Flow cytometric analysis of B cells in the spleen. The frequencies (**c**) and numbers (**d**) of different splenic B cell subsets in WT and *Hnrnpf* KO mice were examined and calculated (*n* = 6 for WT group and *n* = 5 for *Hnrnpf* KO group). Data are representative of five independent experiments (**a** and **c**). Each symbol represents an individual mouse (**b** and **d**). An unpaired two-tailed student’s t-test was used for the statistical analysis (**b** and **d**). Data are presented as mean values ± SD (**b** and **d**). Source data are provided as a Source Data file.

To determine the role of hnRNP F in B cell physiology, we crossed mice harboring *loxP*-flanked (floxed) *Hnrnpf* alleles with *Cd19*^Cre/+^ mice, which specifically express Cre recombinase in B cells, to generate *Hnrnpf *^fl/fl^*Cd19*^Cre/+^ (*Hnrnpf* bKO) mice. We first investigated B cell development in the BM of *Hnrnpf* bKO mice by flow cytometry. Using a gating strategy based on Hardy classification (Supplementary Fig. 1c), we detected indistinguishable pre-pro-B (Fr. A), early pro-B (Fr. B), late pro-B (Fr. C) and large pre-B (Fr. C’, CD24^high(hi)^ BP-1^+^) subsets in the B220^+^CD43^+^ early B cell compartment, as well as IgM^−^IgD^−^ small pre-B (Fr. D), IgM^+^IgD^−^ immature (Fr. E) and IgM^+^IgD^+^ recirculating mature (Fr. F) B cells in the B220^+^CD43^−^ B cell compartment in the BM between *Hnrnpf* ^fl/fl^*Cd19*^+/+^ (WT) and *Hnrnpf* bKO mice (Fig. 1a, b). These results suggest that B cell development in the BM is unaffected in the *Hnrnpf* bKO mice. As previous studies showed that CD19-Cre mediated deletion of some floxed genes was inefficient in early B cell populations in the BM compared to mature B cells in the spleen^28,29^, we next asked if the undisturbed early B lymphopoiesis phenotype in the *Hnrnpf* bKO mice was due to the incomplete ablation of the floxed *Hnrnpf* alleles. We purified various B cell subsets from the BM and spleen of WT and *Hnrnpf* bKO mice and determined *Hnrnpf* mRNA levels by qRT-PCR analysis. We found that CD19-Cre-mediated deletion of *Hnrnpf* gene was only about 20% in Fr. B (early pro-B) cells and the deletion was increased to 50% in the more mature Fr. C (late pro-B), C’ (large pre-B) and Fr. D (small pre-B) cells, and was further improved to 85 and 90% in the Fr. E (immature B) and splenic B cells, respectively (Supplementary Fig. 1d). Immunoblotting further confirmed that hnRNP F protein was barely detected in the *Hnrnpf* bKO splenic B cells (Supplementary Fig. 1e). These results suggest that a more explicit determination of hnRNP F’s role in early B lymphopoiesis requires a more efficient *Hnrnpf* gene deletion in B cells mediated by other Cre-recombinases, such as Mb1-Cre. Next, we examined B cell maturation in the periphery by flow cytometry (Supplementary Fig. 1f). WT and *Hnrnpf* bKO mice manifested indistinguishable B220^+^CD93^+^ transitional B cell subsets (stage I to III) in their spleens (Fig. 1c, d). Interestingly, the *Hnrnpf* bKO mice exhibited an increase in the percentage and absolute number of B220^+^CD93^−^CD23^−^CD21^+^ MZB cells in their spleen compared with the WT mice. Conversely, the percentage of B220^+^CD93^−^CD23^+^CD21^−^ FOB cells was lower than in the WT mice, though the difference in the absolute cell number was not statistically significant (Fig. 1c, d). Thus, these data demonstrate that the hnRNP F-deficient mice have largely unperturbed B cell development except for the slightly increased MZB cell population.

### HnRNP F-deficient mice have a defective TD antibody response

**Fig. 2:**
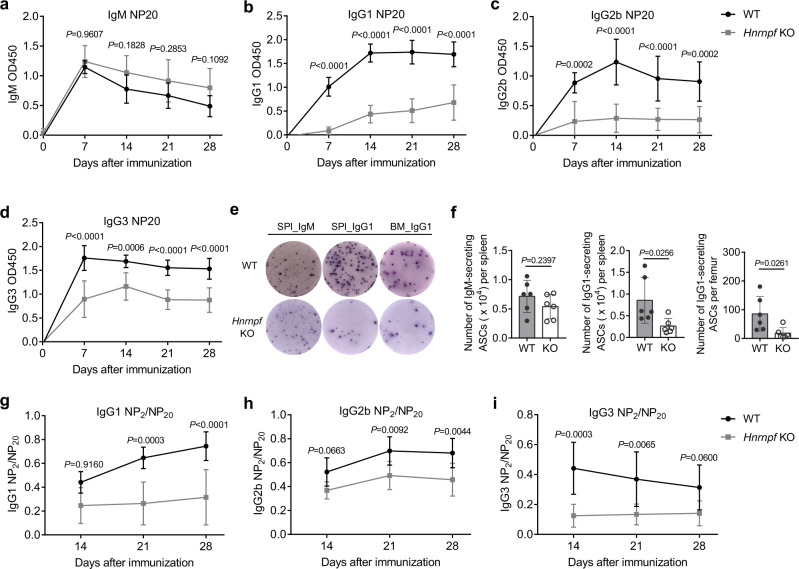
hnRNPF is required for TD antibody immune response. **a**–**d** ELISA of determining antibody titers of NP-specific IgM (**a**), IgG1 (**b**), IgG2b (**c**), and IgG3 (**d**) in the serum of WT and *Hnrnpf* KO mice at various time points post-immunization (*n* = 6 for WT group and *n* = 7 for *Hnrnpf* KO group). **e** ELISPOT assay of NP-specific IgM and IgG1 antibody-secreting cells in the spleen and BM of WT and *Hnrnpf* KO mice at day 10 post-immunization (*n* = 6 per group). **f** Enumeration of antibody-secreting cells in the spleen and BM as determined in **e**. Each symbol represents an individual mouse analyzed. **g**–**i** Ratios of NP-specific IgG1 (**g**), IgG2b (**h**), and IgG3 (**i**) antibody titers as determined with NP_2_-BSA as the coating antigens versus those with NP_20_-BSA as the coating antigens in the sera of WT and *Hnrnpf* KO mice at the various time points post-immunization (*n* = 6 for WT group and *n* = 7 for *Hnrnpf* KO group). Data are from one single experiment (**a**–**d** and **g**–**i**) or representative of six independent experiments (**e**). Two-way ANOVA with Sidak’s multiple comparisons (**a**–**d** and **g**–**i**) or an unpaired two-tailed student’s t-test (**f**) was used for the statistical analysis. Data are presented as mean values ± SD (**a**–**d** and **f**–**i**). Source data are provided as a Source Data file.

We next examined the antibody response in the *Hnrnpf* bKO mice by challenging them with TD antigen 4-hydroxy-3-nitrophenyl-acetyl (NP) hapten conjugated to chicken gamma globulin (CGG). NP-specific IgM and class-switched different isotypes of IgG antibodies in the sera were measured by ELISA at various time points post-immunization. We noticed that *Hnrnpf* bKO mice produced comparable, if not slightly elevated, titers of NP-specific IgM antibodies on par with WT mice across the time points examined (Fig. 2a). In contrast, *Hnrnpf* bKO mice had pronouncedly decreased NP-specific IgG1, IgG2b, and IgG3 antibody titers than the WT mice (Fig. 2b–d). Furthermore, consistent with their normal NP-specific IgM but significantly decreased NP-specific IgG1 antibody titers in the sera, we also detected markedly reduced NP-specific IgG1 antibody-secreting cells (ASC) by ELISPOT assay in the spleen and BM of *Hnrnpf* bKO mice compared to the WT mice (Fig. 2e, f). These results suggest *Hnrnpf* bKO mice have defective class-switched IgG antibody production in response to the TD antigen challenge.

We also performed ELISA to measure the relative changes in antigen-specific serum IgG1, IgG2b, and IgG3 antibody affinity by determining the ratios of antibody titers binding low (NP_2_) to highly (NP_20_) haptenated BSA. We found that the ratios of NP_2_/NP_20_ IgG1, IgG2b and IgG3 titers from *Hnrnpf* bKO mice were significantly lower than those from the WT mice (Fig. 2g–i). These results suggest that *Hnrnpf* bKO mice have compromised antibody affinity maturation during TD immune response.

### Defective IgG1 antibody class-switching in hnRNP F-deficient B cells

**Fig. 3:**
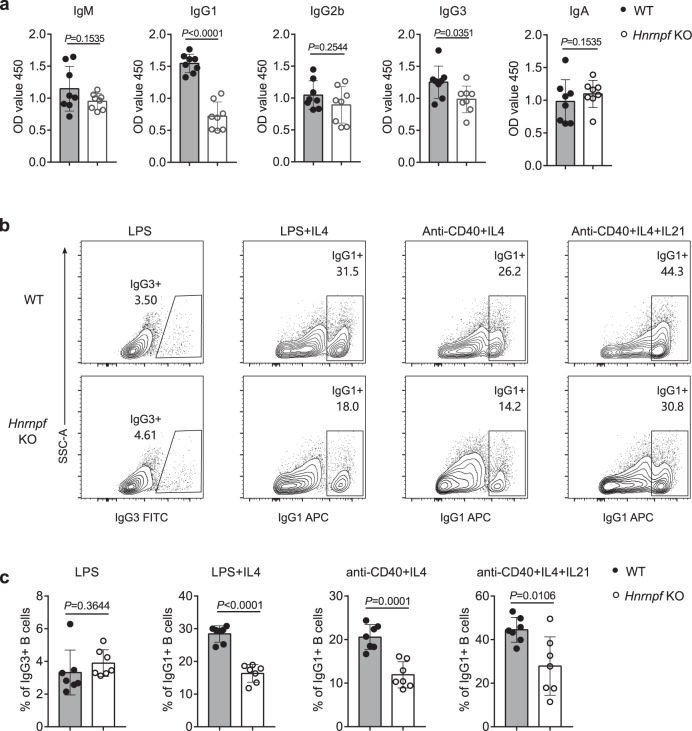
hnRNP F is required for IgG1 CSR. **a** ELISA of determining antibody titers of total IgM, IgG1, IgG2b, IgG3, and IgA in sera of unimmunized WT (*n* = 8) and *Hnrnpf* KO (*n* = 8) mice. Data are from one single experiment. **b**, **c** Flow cytometry measuring CSR to IgG3 or IgG1 in activated B cells with indicated stimulation (*n* = 7 per group). Data are representative of four independent experiments (**b**). An unpaired two-tailed student’s t-test was used for the statistical analysis (**a** and **c**). Data are presented as mean values ± SD (**a** and **c**). Source data are provided as a Source Data file.

The unchanged IgM but decreased class-switched IgG antibody titers suggest that hnRNP F-deficiency may affect antibody class-switch recombination (CSR). Thus, we first measured IgM, IgG1, IgG2b, IgG3, and IgA antibodies in sera from unimmunized WT and *Hnrnpf* bKO mice. Interestingly, we found a significantly reduced IgG1 antibody titer, and a slightly decreased IgG3 antibody level but normal IgG2b and IgA antibody levels in the *Hnrnpf* bKO mice (Fig. 3a). These results suggest that *Hnrnpf* bKO B cells might have intact CSR machinery but are defective in responding to signals that can induce class-switching of IgG1, and IgG3 to a lesser extent.

Next, we examined IgG1 and IgG3 antibody CSR in vitro. We first induced IgG3 antibody CSR by culturing WT and *Hnrnpf* bKO splenic B cells in the presence of LPS for three days, followed by flow cytometric analysis. Equivalent percentages of IgG3^+^ cells were detected in the WT and *Hnrnpf* bKO B cell culture (Fig. 3b, c). In contrast, the IgG1 antibody CSR was severely compromised in the absence of hnRNP F when B cells were cultured with LPS and interleukin 4 (IL4), anti-CD40 antibody and IL4, and anti-CD40 antibody, IL4, and IL21, though the addition of IL21 seemed to be able to partially improve IgG1 antibody CSR in the mutant B cells (Fig. 3b, c). These results suggest that hnRNP F is essential for IgG1 antibody CSR.

### Defective GC B cell response in *Hnrnpf* bKO mice

**Fig. 4:**
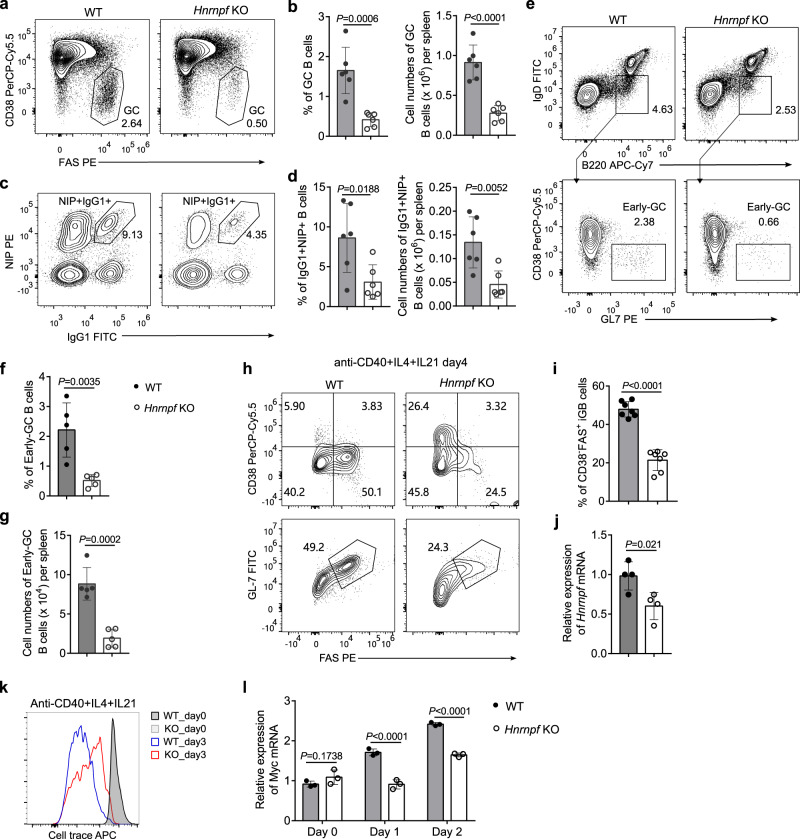
hnRNP F is essential for GC B cell formation. **a**, **b** Flow cytometric analysis of the frequencies (**a**) and numbers (**b**) of total GC B cells in the spleen of WT and *Hnrnpf* KO mice (*n* = 6 per group) at day 10 post-immunization. **c**, **d** Flow cytometric analysis of the NP-specific IgG1^+^ B cells in the spleen of WT and *Hnrnpf* KO mice (*n* = 6 per group) at day 10 post-immunization. NP-specific IgG1^+^ B cells were determined by IgG1 and NIP^+^ BCR expression on the B220^+^Dump (CD138, IgM, CD3, IgD, Gr1)^-^ cells (**c**). The frequencies and numbers of NP-specific IgG1^+^ B cells (**d**) were calculated accordingly. **e**–**g** Flow cytometric analysis of early GC B cell formation in the spleen of WT and *Hnrnpf* KO mice (*n* = 5 per group) at day 5 post-immunization. B220^+^GL-7^+^IgD^-^CD38^-^ GC B cells were determined by flow cytometry (**e**), and their frequencies (**f**) and numbers (**g**) were summarized. **h**–**j** Flow cytometric analysis of iGB cells induced by anti-CD40, IL4, and IL21 for 4 days. B220^+^GL-7^+^Fas^+^CD38^-^ iGC B cells were determined by flow cytometry (**h**), and their frequencies (**i**) and the *Hnrnpf* deletion efficiency (**j**) were summarized (*n* = 7 per group in (**i**) and *n* = 4 per group in **j**). **k** Analysis of proliferation of B cells stimulated with iGB condition for 3 days using cell trace dye. **l** qRT-PCR analysis of Myc expression in purified splenic B cells stimulated with iGB condition for 2 days. Data are representative of six independent experiments (**a** and **c**), three independent experiments (**e**, **j**, and **k**), four independent experiments (**h**), or one single experiment (**l**). Each symbol represents an individual mouse (**b**, **d**, **f**, **g**, **i**, **j**, and **l**). An unpaired two-tailed student’s t-test (**b, d, f, g, i,** and **j**) or two-way ANNOVA with Sidak’s multiple comparisons (**l**) was used for the statistical analysis. Data are presented as mean values ± SD (**b**, **d**, **f**, **g**, **i**, **j,** and **l**). Source data are provided as a Source Data file.

During TD antibody response, the class-switched antigen-specific antibodies with high affinities are produced via GC response^30^. Given the impaired production of antigen-specific antibodies and defective IgG1 antibody CSR in *Hnrnpf* bKO mice, we sought to examine the GC response in detail in the mutant mice. We first challenged WT and *Hnrnpf* bKO mice with NP-CGG followed by an assessment of the peaked GC B cell response 10 days post-challenge by flow cytometry (Supplementary Fig. 1g, h). We found that the frequency and number of B220^+^Fas^+^CD38^−^ GC B cells were severely reduced by more than 70% in the spleen of *Hnrnpf* bKO mice compared to the WT mice (Fig. 4a, b). We also noticed that the percentage and number of NP-specific IgG1^+^NIP^+^ B cells were also significantly lower in *Hnrnpf* bKO than in the WT mice (Fig. 4c, d). These results indicate that the GC B cell response is severely compromised in *Hnrnpf* bKO mice.

To gain more insights into how hnRNP F-deficiency affected GC B cell response, we further examined the distribution of CXCR4^high(hi)^ CD83^low(lo)^ DZ and CXCR4^lo^CD83^hi^ LZ B cells in the GC B cell compartment by flow cytometry. To this end, we first gated NP^+^ cells, which were genuine activated B cells upon the NP-CGG immunization, followed by the analysis of DZ and LZ B cells in WT and *Hnrnpf* bKO (Supplementary Fig. 2a). Surprisingly, we found that the ratios of NP-specific DZ to LZ B cells were equivalent between the WT and *Hnrnpf* bKO mice, albeit the percentage of the total NP^+^ GC B cells was largely reduced in the *Hnrnpf* bKO mice (Supplementary Fig. 2a, b). However, when we examined the *Hnrnpf* mRNA levels in the GC B cells from the *Hnrnpf* bKO and WT mice, we found that they were largely comparable (Supplementary Fig. 2c). We reasoned that the GC B cells in the *Hnrnpf* bKO mice were most likely from an expansion of the hnRNP F-proficient GC B cells differentiating from the residual FOB cells that have escaped from CD19-Cre-mediated *Hnrnpf* deletion.

Next, we attempted to understand how hnRNP F-deficiency caused the saliently diminished GC B cells by examining the early GC B cell response when the nascent GC B cells were just formed (Supplementary Fig. 1i). We noticed that *Hnrnpf* bKO mice manifested a drastically reduced B220^+^IgD^−^GL-7^+^CD38^−^ early GC B cell population in the spleen on day 5 post-immunization (Fig. 4e, f). Enumeration of these early GC B cells revealed that the early GC B cell population was dramatically diminished in the *Hnrnpf* bKO mice as their absolute number was decreased by about 80% compared to the WT mice (Fig. 4g). These data suggest that the generation of GC B cells is severely compromised in the absence of hnRNP F. We also set up an in vitro induction of GC-phenotype B (iGB) cells as described previously^31^. Consistent with the results of early GC B cells, the generation of iGB cells was reduced by approximately 50% compared to the WT cells (Fig. 4h, i). Intriguingly, the mRNA level of *Hnrnpf* in iGB culture using the mutant B cells was about 60% of that in the culture using the WT cells (Fig. 4j), much higher than that in the freshly purified and yet-to-be-differentiated *Hnrnpf* bKO B cells, which was only 10% of the level in the WT B cells (Supplementary Fig. 1d). One of the possible reasons for the drastically different mRNA levels of *Hnrnpf* in mutant B cells before and after iGB differentiation could be the hnRNP F-deficient early differentiating GC B cells were defective in proliferation, such that they were much less representative compared to the deletion-escaped hnRNP F-proficient differentiating GC B cells that can proliferate normally. To test this possibility, we cultured WT and mutant B cells labeled with cell tracer under the iGB differentiation condition and examined the proliferation by flow cytometry. It was revealed that *Hnrnpf* bKO B cells were defective in cell proliferation (Fig. 4k). It is known that transcriptional factor c-Myc is induced during and necessary for GC B cell response^32,33^. We next examined the *c-Myc* expression by qRT-PCR and found that the upregulation of *c-Myc* was severely compromised in cultured *Hnrnpf* bKO B cells than their WT counterparts (Fig. 4l). Collectively, these results suggest that hnRNP F plays an essential role in regulating GC B cell formation by promoting early GC B cell proliferation, which requires c-Myc upregulation.

### HnRNP F controls AS of *Cd40* pre-mRNA in FOB cells

FOB cells are the prototypic B cell subset that responds to TD antigen mounting robust antibody response through GC reaction^34,35^. Given that hnRNP F-deficient mice manifested a slightly decreased percentage of FOB cells and drastically compromised early GC B cell formation, we hypothesized that hnRNP F could be required to maintain FOB cells and their subsequent activation and differentiation into GC B cells. Thus, we performed RNA sequencing (RNA-Seq) on FACS-sorted WT and *Hnrnpf* bKO FOB cells. Analysis of RNA-Seq data revealed that mRNA abundances of 157 genes were significantly increased and that of 79 genes were decreased (FDR (false discovery rate) ≤ 0.05, fold change ≥ 2) in *Hnrnpf* bKO FOB cells compared to their WT counterparts (Supplementary Fig. 3a and Supplementary Data 1). We further performed a Kyoto Encyclopedia of Genes and Genomes (KEGG) pathway enrichment analysis of the differentially expressed genes. Interestingly, PI3K-Akt and MAPK signaling pathways were identified as the top two pathways showing enrichment for genes differentially expressed in hnRNP F-deficient FOB cells (Supplementary Fig. 3b). These data imply that hnRNP F is essential for regulating the expression of genes encoding molecules crucially involved in PI3K-Akt and MAPK signaling pathways in FOB cells.

**Fig. 5:**
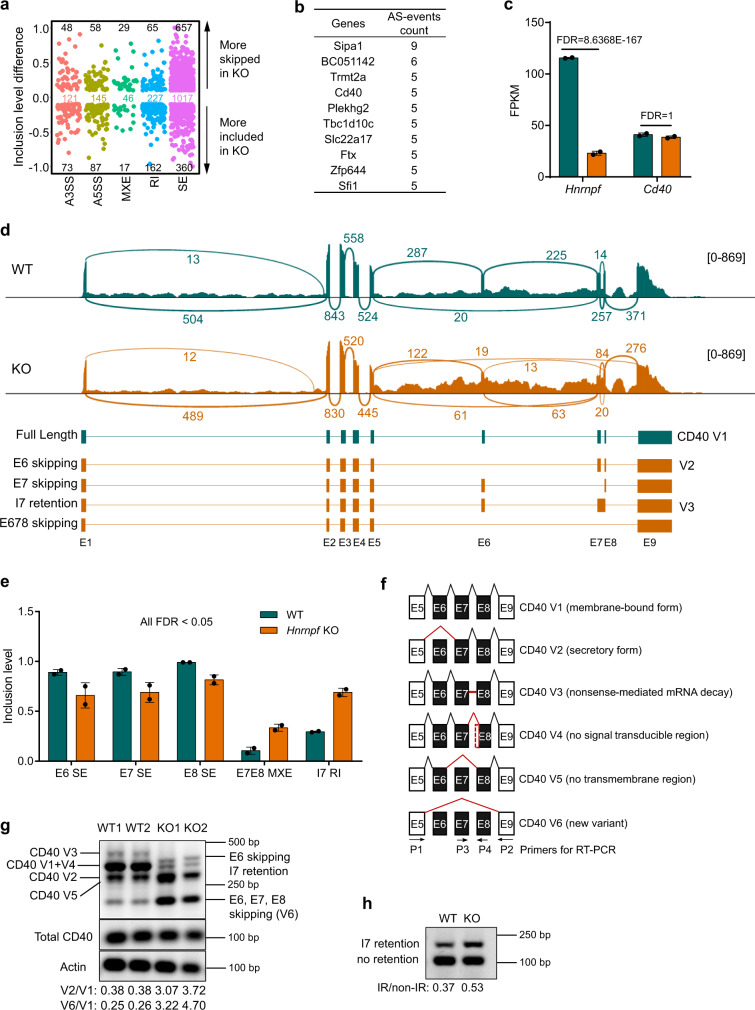
hnRNP F controls *Cd40* AS. **a** Scatter plots of inclusion level difference between WT and *Hnrnpf* KO FOB cells. Each dot represents a significantly altered splicing event (|inclusion level difference | > 10%, FDR < 0.05). **b** A list of top ten genes with the highest aberrant AS events. **c** Relative expression of *Hnrnpf* and *Cd40* mRNA in FOB cells based on the RNA-seq data (*n* = 2 per group). Data are from one single experiment. FDR was calculated by DESeq2 Bioconductor package based on the negative binomial distribution and and Benjamini–Hochberg method. **d** Sashimi plot of changes in AS of *Cd40* exon 6-8 in WT and *Hnrnpf* KO FOB cells. The full-length version and different variants of *Cd40* were shown. **e** The comparison of inclusion levels of indicated *Cd40* AS events between WT and *Hnrnpf* KO FOB cells. FDR was calculated by rMATS. Likelihood-ratio test was used to calculate *p* value of the Inclusion Level between two groups. Benjamini Hochberg algorithm was used to correct *p* value to get FDR value (*n* = 2 per group). Data are from one single experiment. **f** Schematics of various isoforms of *Cd40* mRNA generated by AS. The red lines above the exon indicate exon junctions, and the red line in CD40 V3 indicates CD40 intron 7. The red dotted line indicates exon sequences that are missing in CD40 V4 compared to CD40 V1. The arrows indicate the position of PCR primers for measuring *Cd40* AS. **g** RT-PCR analysis of *Cd40* variants using primers P1 and P2. The ratios of CD40 V2 to V1 and V6 to V1 were shown below the gel image. **h** RT-PCR analysis of *Cd40* intron 7 retention using primers P3 and P4. The ratio of the variant with I7 retention to that without was shown below the gel image. Data are representative of three independent experiments (**g**) or two independent experiments (**h**). Data are presented as mean values ± SD (**c** and **e**). Source data are provided as a Source Data file.

Since hnRNP F’s primary function in the post-transcriptional gene regulation is to modulate AS of pre-mRNAs^5,36–38^, we next focused on studying differential AS events between the WT and *Hnrnpf* bKO FOB cells using rMATS, a robust computational tool for detecting differential AS events from the RNA-Seq data^39^. Among the five types of AS events, exon skipping (SE) is the most affected by hnRNP F-deficiency, with 657 events more skipped in *Hnrnpf* bKO than in the WT B cells and 360 events more included in *Hnrnpf* bKO than in the WT B cells (Fig. 5a and Supplementary Data 2). This result is consistent with previous reports showing that hnRNP F mainly regulates exon skipping or inclusion^6,8,13,37,40^. Among the top 10 genes with the highest aberrant AS events, the *Cd40* gene drew our attention (Fig. 5b), given that CD40 is critical for antibody CSR, c-Myc induction, and GC B cell proliferation^24,41^. However, we noticed that the abundance of *Cd40* mRNA was not significantly affected in the mutant FOB cells (Fig. 5c). As presented in sashimi plots, exon junction visualization revealed that hnRNP F predominantly controls AS of *Cd40* pre-mRNA from exon 6 to 9 but not from exon 1 to 5 (Fig. 5d). Further analysis revealed multiple aberrant *Cd40* AS events in *Hnrnpf* bKO FOB cells, including skipped exon (SE) 6, 7, and 8, mutually exclusive exon (MXE) 7 and 8, and retained intron (RI) 7 (Fig. 5e).

To validate *Cd40* AS changes in hnRNP F-deficient FOB cells, we performed RT-PCR and Sanger Sequencing to examine the abundance of various *Cd40* mRNA variants using previously reported primers (Fig. 5f), which can cover exons 5–9^42^. We showed that *Cd40* pre-mRNA in WT FOB cells had an AS pattern consistent with the previous reports^42^. As the sizes of variant 1 (V1) and V4 PCR products were very similar and could not be distinguished readily by the electrophoresis, we performed Sanger sequencing and showed that V1, the membrane-bound and signaling-competent form, but not V4, the signaling-incompetent form of *Cd40*, was predominant in both WT and KO B cells (Supplementary Fig. 3c). Interestingly, the loss of hnRNP F resulted in a drastic decrease in *Cd40* V1 accompanied by a significant increase in *Cd40* V2, as evidenced by the markedly increased V2/V1 ratio (Fig. 5g). We also detected two previously unidentified variants, one with exon 6 skipped and intron 7 retention and the other one with exons 6, 7 and 8 all skipped. Interestingly, a small amount of the variant with exons 6, 7 and 8 skipping was also present in the WT B cells, but it had not been reported previously (Fig. 5g). Thus, we named it V6. As the intron 7 retention could not be well-discerned in the PCR products amplified with primers P1 and P2, we used primers P3 and P4 to further evaluate the proportion of intron 7 retention. A higher ratio of variants with intron 7 retention to that with no retention was detected in *Hnrnpf* bKO B cells compared with the WT B cells (Fig. 5h). Together these results suggest that hnRNP F regulates the AS of *Cd40* pre-mRNA by promoting exons 6, 7, and 8 inclusion and intron 7 removal.

A previous study of B cell lines showed that hnRNP F promotes the membrane-bound IgM (mIgM) expression and inhibits the secretory type IgM (sIgM) production through regulating APA of IgH chain mRNA^14^. Thus, we also compared the expression of mIgM on the surface of total splenic B cells and FOB cells from the WT and *Hnrnpf* bKO mice by flow cytometry and found them to be comparable (Supplementary Fig. 3d, e). Moreover, the analysis of RNA sequencing data also indicated that mIgM-encoding transcript was predominantly and equivalently present in WT and *Hnrnpf* KO FOB cells, and they barely had any sIgM-encoding transcript (Supplementary Fig. 3f). These results suggest hnRNP F-deficiency does not affect APA of IgH chain mRNA in mouse splenic B cells.

### HnRNP F regulates GC B cell formation by promoting signaling-competent CD40 expression

**Fig. 6:**
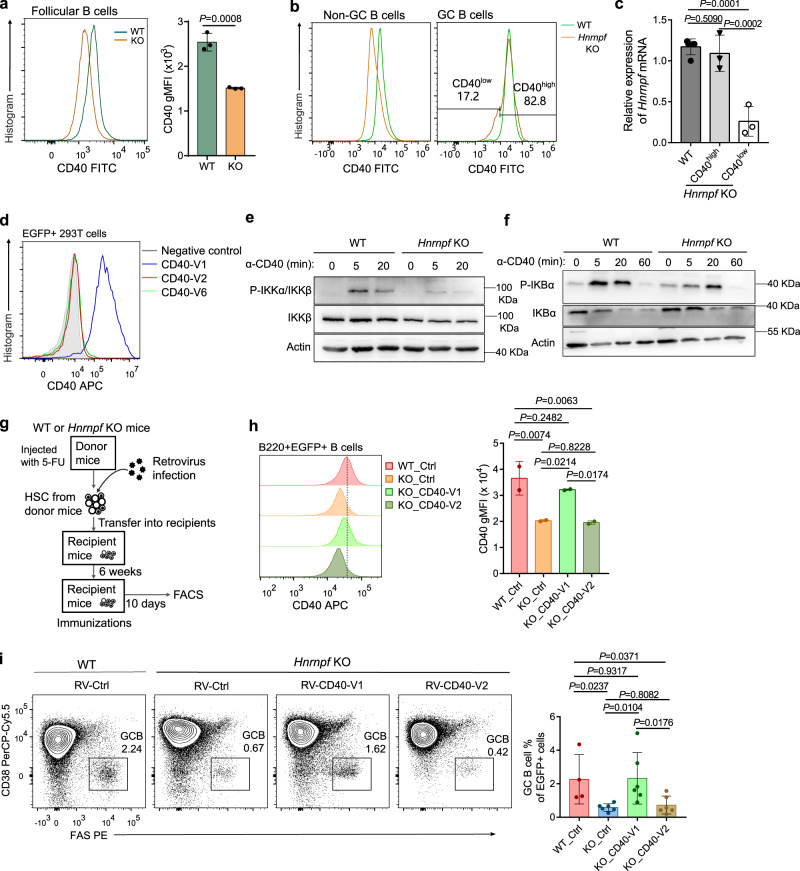
hnRNP F regulates GC B cell formation by promoting signalling-competent CD40 expression. **a** Flow cytometric analysis of the cell surface expression of CD40 on WT and *Hnrnpf* KO FOB cells (*n* = 3 per group). **b** Flow cytometric analysis of the cell surface expression of CD40 on WT and *Hnrnpf* KO GC (B220^+^CD38^-^FAS^+^) and non-GC (B220^+^CD38^+^FAS^-^) B cells. **c** Knockout efficiency of hnRNP F in *Hnrnpf* KO GCB cells with high and low CD40 expression using qRT-PCR (*n* = 4 for WT group and *n* = 3 for *Hnrnpf* KO group). **d** Flow cytometry analysis of the cell surface expression of CD40 on 293 T cells transfected with different CD40 variants. **e**, **f** Immunoblotting of phosphorylation of IKKα/IKKβ (**e**) and IκBα (**f**) in WT and *Hnrnpf* KO splenic B cells stimulated by 1 μg/ml anti-CD40 antibody. Data are from one single experiment (**e** and **f**). **g** The experimental flow of mixed BM chimeric mice study. **h** Flow cytometric analysis of the cell surface expression of CD40 on splenic B cells from different chimeric mice as indicated at day 10 after immunization with NP-CGG (*n* = 2 per group). **i** Flow cytometric analysis of GC B cells in the spleen of chimeric mice at day 10 after immunization (*n* = 4 for WT group and *n* = 6 for *Hnrnpf* KO group). Data are representative of two independent experiments (**a**, **b,** and **d**) or three independent experiments (**h** and **i**). Each symbol represents an individual mouse, and an unpaired two-tailed student’s t-test (**a**) or one-way ANOVA without adjustments (**c**, **h,** and **i**) was used for the statistical analysis. Data are presented as mean values ± SD (**a**, **c**, **h,** and **i**). Source data are provided as a Source Data file.

Given the dramatically altered AS of *Cd40* mRNA in *Hnrnpf* bKO FOB cells (Fig. 5), we asked if the cell surface expression of CD40 was affected in the mutant cells. Indeed, flow cytometric analysis revealed that FOB cells from the unimmunized *Hnrnpf* bKO mice expressed much lower CD40 on their cell surface than the cells from the WT mice (Fig. 6a). Next, we examined the cell surface expression of CD40 in B cells from immunized mice. Similar to the situation of resting FOB cells, the CD38^+^Fas^−^ non-GC B cells, supposedly non-dividing cells, from *Hnrnpf* bKO mice expressed much lower levels of CD40 than their WT counterparts (Fig. 6b). However, consistent with the largely normal expression of *Hnrnpf* (Supplementary Fig. 2c), the residual CD38^−^Fas^+^ GC B cells from the *Hnrnpf* bKO mice had similar levels of CD40 compared to their counterparts from the WT mice, except for a small fraction (~15-20%) of GC B cells expressed lower levels of CD40 on their cell surface (Fig. 6b). We reasoned that the majority of residual GC B cells in immunized *Hnrnpf* bKO mice was from the B cells that escaped *Hnrnpf*-deletion and underwent expansion, thus having normal CD40 expression. Only a small fraction of GC B cells were the genuine hnRNP F-deficient cells, and hence, had lower CD40 expression. Thus, we sorted the CD40^hi^ and CD40^lo^ GC B cells from the immunized *Hnrnpf* bKO mice and examined the *Hnrnpf* expression by qRT-PCR. It was corroborated that the CD40^lo^ GC B had much lower levels of *Hnrnpf* mRNA than their CD40^hi^ counterparts and WT GC B cells (Fig. 6c).

To further evaluate the cell surface expression of *Cd40* V2 and V6, the two most dominant variants present in *Hnrnpf* bKO FOB cells, we transfected HEK 293 T cells with plasmids expressing these two variants. In contrast to the ready detection of CD40 on the 293 T cells transfected with a full-length *Cd40* V1, CD40 was barely detected on the cell surface of 293 T cells over-expressing V2 and V6 (Fig. 6d). These results are consistent with the previous study reporting that only *Cd40* V1 but not the exon 6-skipped variants, such as V2 and V6, can be translated into a membrane-bound CD40 receptor^42^. We also assessed the biochemical consequence of the decreased cell surface expression of CD40 in the mutant B cells by examining the activation of NF-κB, which was picked up by the gene enrichment analysis (Supplementary Fig. 3b) and is one of the most important signaling pathways downstream of CD40 signaling^43^. Our immunoblotting showed that hnRNP F-deficient splenic B cells exhibited much reduced NF-κB activation upon stimulation with an agnostic anti-CD40 antibody, as evidenced by their significantly decreased phosphorylation of IKKα/β and their substrate IKBα compared to the WT B cells (Fig. 6e, f). These results also dovetailed with the previous data that hnRNP F-deficient B cells were defective in proliferation upon treatment with anti-CD40 antibody plus IL-4, and IL-21, and failed to upregulate c-Myc upon CD40 engagement (Fig. 4l). Our results suggest that the altered *Cd40* AS due to hnRNP F-deficiency leads to significantly reduced cell surface expression of CD40, and consequently compromised CD40-mediated downstream signaling in B cells.

Next, we asked if the compromised GC B cell formation in *Hnrnpf* bKO mice was due to the defective cell surface expression of CD40 caused by the abnormal *Cd40* mRNA AS in the mutant B cells. To this end, we performed a mixed BM chimera experiment by transferring 33.3% of *Hnrnpf* bKO BM cells transduced retrovirally with V1 or V2 *Cd40* variant together with 66.6 % of BM cells from the μMT mice into the sub-lethally irradiated WT mice. The chimeric mice were challenged with NP-CGG 6 weeks after reconstitution, and GC B cells were analyzed 10 days after immunization (Fig. 6g and Supplementary Fig. 4a). Flow cytometric analysis showed that the defective cell surface expression of CD40 in the hnRNP F-deficient B cells was largely restored by *Cd40* V1 but not by the V2 variant (Fig. 6h). More importantly, the reintroduction of *Cd40* V1 variant into the hnRNP F-deficient B cells could largely rescue their defective GC B cell formation (Fig. 6i). In contrast, the *Cd40* V2 variant failed to salvage the GC B cell defect of *Hnrnpf* bKO mice. Examination of *Hnrnpf* transcript further showed that the GC B cells differentiated from the *Hnrnpf* bKO B cells rescued by *Cd40* V1 variant had lower levels of *Hnrnpf* mRNA (~ 50% of the levels in WT), suggesting a considerable fraction of GC B cells in these mice were from the *Hnrnpf*-deleted B cells, whereas the residual GC B cells from the *Hnrnpf* bKO cells rescued with the V2 variant had similar *Hnrnpf* mRNA levels compared to the unrescued control cells (~ 80 %), indicating they were most likely from the deletion-escaped cells (Supplementary Fig. 4b). These results suggest the V1 but not the V2 variant of *Cd40* can partially rescue the GC B cell defects caused by hnRNP F-deficiency. Consistently, the abundance of NIP^+^IgG1^+^ cells also increased after the *Cd40* V1 variant rescue, although the difference was not statistically significant (Supplementary Fig. 4c, d). Thus, our results indicate that hnRNP F regulates GC B cell formation, at least partially, by ensuring a proper cell surface expression of CD40 via regulating AS of *Cd40* pre-mRNA.

### HnRNP F directly binds to G-tracts of *Cd40* pre-mRNA to modulate exon inclusion and intron removal

**Fig. 7:**
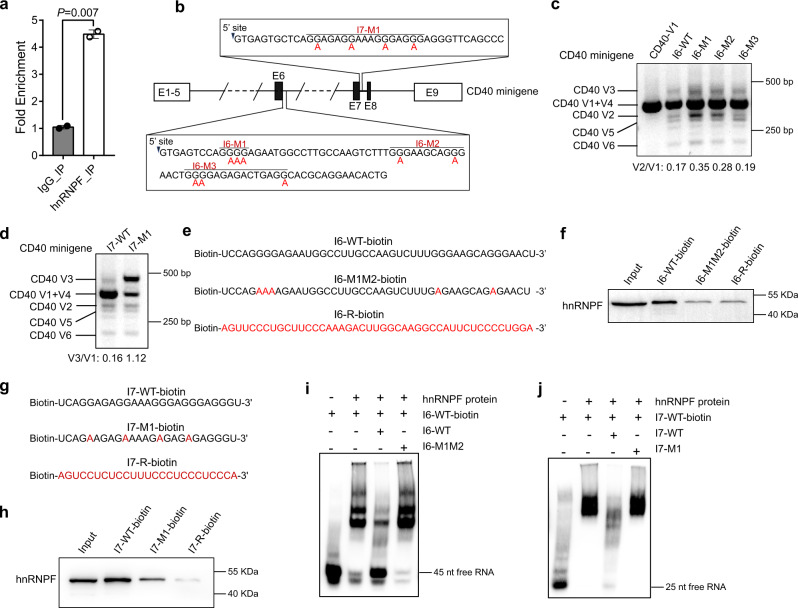
hnRNP F directly binds to G-tracts of *Cd40* pre-mRNA to modulate exon inclusion and intron removal. **a** RIP-qPCR analysis of the enrichment of *Cd40* gene bound to hnRNP F in WT splenic B cells (*n* = 2). Data are from one single experiment. An unpaired two-tailed Welch’s t test was used for statistical analysis. Data are presented as mean values ± SD. **b** Illustration of *Cd40* minigenes. Filled boxes: alternatively spliced exons. Empty boxes: constitutively spliced exons. The mutated bases were highlighted in red. **c**, **d** RT-PCR analysis of *Cd40* variants, including exon 6 skipping (**c**) and intron 7 retention (**d**), using primer P1 and P2 in 3T3-L1 cells transfected with WT or mutated *Cd40* minigenes as indicated. CD40 V1 was used as a positive control. The ratios of CD40 V2 to V1 and V3 to V1 were shown below the gel images. **e**–**h** RNA pull-down assays using biotin-labeled RNAs. Biotinylated RNAs, containing WT or mutated intron 6 (**e**) or 7 (**g**) sequences of the *Cd40* pre-mRNA were prepared. The red letters indicate the mutant bases. RNAs with the reverse sequences were included as controls. The precipitated hnRNP F proteins were examined by immunoblotting (**f** and **h**). **i**, **j** RNA EMSA detection of hnRNP F binding to intron 6 (**i**) and intron 7 (**j**) of *Cd40* pre-mRNA. Data are representative of two independent experiments (**c**, **d**, **i,** and **j**) or three independent experiments (**f**, **h**). Source data are provided as a Source Data file.

We subsequently explored the molecular mechanisms whereby hnRNP F regulates the AS of *Cd40*. We performed a native RNA immunoprecipitation (RIP) using a hnRNP F-specific antibody followed by qRT-PCR analysis to examine hnRNP F’s binding of *Cd40* pre-mRNA in FOB cells. First, a pair of primers specific to *Cd40* exon 6 was used to test the pull-down efficiency of the anti-hnRNP F antibody. A significantly higher amount of *Cd40* mRNA was detected in the RNA precipitated by the anti-hnRNP F antibody than that pulled down by a control IgG antibody (Fig. 7a), suggesting that the antibody can specifically enrich hnRNP F-associated *Cd40* mRNA in B cells.

Next, we set to identify hnRNP F’s binding sites within the *Cd40* locus critical for the AS of *Cd40*. Previous studies have demonstrated that hnRNP F can bind to the G-rich motif within G-tracts in the vicinity of target exons, thus regulating their inclusion or skipping^9,36^. By analyzing introns 6 and 7 sequences adjacent to exons 6 and 7, we found many G-tracts adjacent to the 5’ splicing sites of introns 6 and 7, implying that they might be the potential hnRNP F’s binding sites and required for the AS of *Cd40*. To validate if these putative sites are essential for the normal AS of *Cd40*, we first performed a minigene-based splicing assay. We constructed a WT *Cd40* minigene containing partial sequences of *Cd40* introns 5 and 6, with most of their central regions deleted, plus the intact introns 7 and 8 and exons 1 to 9 of *Cd40* (Fig. 7b). We also made several mutant *Cd40* minigenes with G mutated into A in the G-tract regions 1, 2, and 3 of intron 6 and region 1 of intron 7, namely I6-M1, I6-M2, I6-M3 and I7-M1 (Fig. 7b, shown in the boxes). The mouse 3T3-L1 cells were then transfected with the WT or various mutant minigenes, and RNAs were extracted after 48 h and subjected to qRT-PCR analysis using primers P1 and P2. We found that cells transfected with I6-M1 or I6-M2 minigene expressed considerably higher levels of *Cd40* V2 transcript and increased V2 to V1 ratios (Fig. 7c, lanes 3 and 4). These results were in great contrast to that of cells transfected with I6-WT minigene, which predominantly expressed *Cd40* V1 transcript and very little *Cd40* V2 transcript and thus had a low V2 to V1 ratio (Fig. 7c, lane 2). Conversely, levels of *Cd40* V2 transcript and V2 to V1 ratios were indistinguishable between I6-WT and I6-M3 transfectants (Fig. 7c, lanes 2 and 5). These results suggest that the G-tracts in regions 1 and 2 but not 3 of intron 6 are important for exon 6 inclusion. We also noticed that I7-M1 transfectants manifested a substantially enhanced level of *Cd40* V3 transcript and a much higher V3 to V1 ratio than I7-WT transfectants (1.12 vs 0.16) (Fig. 7d), indicating that G-tracts in this region of intron 7 are critical for preventing I7 retention. Together, our results suggest that G-tracts within intron 6 regions 1 and 2 promote exon 6 inclusion, whereas the G-tracts in intron 7 region 1 facilitate its removal.

We next asked if the above-verified G-tracts are the bona fide binding sites for hnRNP F. To this end, we performed an RNA pull-down assay using biotin-tagged synthesized WT (I6-WT or I7-WT) or mutant RNAs covering regions 1 and 2 of intron 6 (I6-M1M2) or region 1 of intron 7 (I7-M1), respectively (Fig. 7e, g), followed by immunoblotting to detect the hnRNP F protein pulled down by the RNA. We noticed that hnRNP F protein was readily detected in the lysates pulled down by the biotinylated I6-WT or I7-WT RNAs but not by their reverse sequences (I6-reverse and I7-reverse) (Fig. 7f, h, lanes 2 and 4). However, the amount of hnRNP F protein was much lower in the lysate pulled down by the I6-M1M2 or I7-M1 RNA sequences (Fig. 7f, h, lane 3), suggesting that these G-tract regions are the authentic hnRNP F-binding sites.

To further confirm if hnRNP F could directly bind to these G-tract RNA sequences of *Cd40*, we performed an RNA electrophoretic mobility shift assay (EMSA). To this end, His-tagged hnRNP F protein was used to determine the interaction between protein and *Cd40* I6 G-tract RNA. The addition of hnRNP F caused a pronounced retarded migration of the I6-WT-biotin RNA (Fig. 7i, lane 2), compared to the fast migration of the RNA-alone sample (Fig. 7i, lane 1). The retarded migration of I6-WT-biotin RNA by hnRNP F could be partially reversed by the addition of unlabeled I6-WT RNA, which could compete for binding with hnRNP F (Fig. 7i, lane 3) but not by the unlabeled mutant form of I6 RNA, I6-M1M2 (Fig. 7i, lane 4). These results substantiate that hnRNP F directly binds to *Cd40* I6 at regions 1 and 2. We further performed a similar assay to examine the binding of hnRNP F to the *Cd40* I7 region and corroborated that hnRNP F also directly binds *Cd40* I7 at region 1 (Fig. 7j).

### HnRNP A1/A2B1 and hnRNP F antagonistically regulate the AS of *Cd40* RNA

**Fig. 8:**
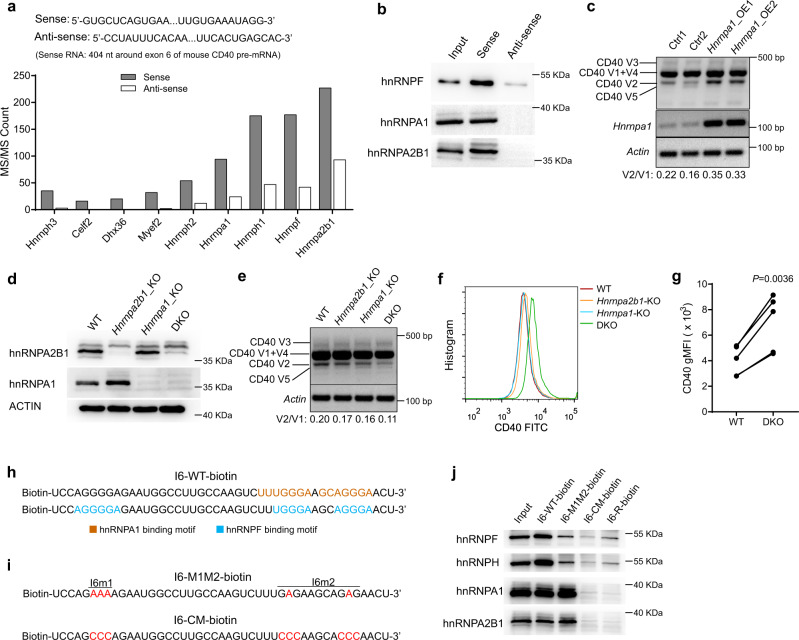
hnRNP A1 and A2B1 bind directly to *Cd40* pre-mRNA and regulate its AS. **a** *Cd40* pre-mRNA-binding proteins pulled down by the sequence corresponding its exon 6 and the flanking intronic regions. The pulled-down proteins were analyzed by mass spectrometry. Top proteins with a fold change of LFQ intensity of more than 5 and MS/MS count with sense RNA of more than 15 were shown. Data are from one single experiment. **b** Immunoblotting of hnRNP F, A1, and A2B1 proteins in the pull-down lysates using biotinylated sense and antisense RNAs as shown in **a**. Data are representative from two independent experiments. **c** RT-PCR analysis of *Cd40* variants using primers P1 and P2 in B cells retrovirally transduced with control or hnRNP A1-overexpressing vectors. **d** Immunoblotting of hnRNP A1 or A2B1 in the splenic B cells from the WT and different knockout mice. **e** RT-PCR analysis of *Cd40* variants using primers P1 and P2 in the FOB cells from the WT and different knockout mice. **f**, **g** Flow cytometric (**f**) and statistical analysis (**g**) of the cell surface expression of CD40 expression in FOB cells from WT and DKO mice. Data are pooled from three independent experiments in which the pairing samples were the mice from the same litters (*n* = 5 per group). A paired two-tailed student’s t-test was used for the statistical analysis. **h** The predicted binding sites of hnRNP F and hnRNP A1 on *Cd40* I6-WT RNA by RBPmap. **i**, **j** RNA pull-down assays using WT or indicated mutated biotin-labeled RNAs (**i**), and the precipitated hnRNP proteins were examined by immunoblotting (**j**). Data are representative of two independent experiments (**c**–**e**) or three independent experiments (**j**). Source data are provided as a Source Data file.

More than half of the AS events are regulated by complexes of multiple hnRNP proteins, acting either synergistically or antagonistically^38,44^. Having demonstrated the critical role of hnRNP F in *Cd40* AS, we next asked if other hnRNPs also regulate the AS of *Cd40* given that CD40 is crucial for B cell physiology. To this end, we performed an RNA pull-down assay followed by protein mass spectrometry to identify the potential hnRNP proteins involved in regulating the AS of *Cd40*. A sense RNA of 404 nucleotides in length, including the exon 6 and the adjacent region of intron 6 sequences of mouse *Cd40* pre-mRNA, was transcribed in vitro by the T7 RNA polymerase and labeled with biotin. An antisense RNA of the same region was prepared and served as a control. Mass spectrometric analysis of proteins pulled down by the sense and antisense RNA identified nine proteins with fold-change (sense vs antisense pull-down) of label-free quantification (LFQ) more than 5. Six of the nine proteins are hnRNP proteins, including hnRNP H1, hnRNP H2, hnRNP H3, hnRNP F, hnRNP A1, and hnRNP A2B1(Fig. 8a). Among the six hnRNP proteins, hnRNP H1, hnRNP H2, hnRNP H3, and hnRNP F belong to the same hnRNP F/H subfamily and contain the q-RRMs for recognizing G-tracts and regulating AS^9,10,45^. Our EMSA demonstrated that hnRNP H1 could bind to the same sites of *Cd40* pre-mRNA as hnRNP F did (Supplementary Fig. 5a, b). Moreover, our pull-down assay also identified CELF2, a homolog of CELF1 that was previously shown to promote *Cd40* exon 6 inclusion in human B lymphoma cell line Daudi^27,46^, to bind the same region of *Cd40* pre-mRNA, further proving the fidelity of our assay (Fig. 8a).

Interestingly, the most abundant protein identified in the pull-down experiment was hnRNP A2B1, with its homolog A1 also on the list. HnRNP A1 and A2B1 are members of hnRNPs A/B subfamily and can bind G/A-rich sequences to regulate AS of RNAs^38,47,48^. Consistently, our immunoblotting assay detected the presence of hnRNP A1 and A2B1 in a sense but not antisense RNA pull-down lysates (Fig. 8b), corroborating that these two hnRNP proteins bind to *Cd40* pre-mRNA sequences adjacent to exon 6 as hnRNP F does. To further explore the role of hnRNP A1 and A2B1 in regulating AS of *Cd40*, we retrovirally overexpressed *Hnrnpa1* and *Hnrnpa2b1*, respectively, in mouse splenic B cells. Our RT-PCR analysis showed that hnRNP A1 overexpression led to significantly increased levels of *Cd40* V2 transcript compared to the control group (Fig. 8c). These results suggest that hnRNP A1 promotes *Cd40* RNA exon 6 skipping, consistent with previous data that hnRNPA1 mainly promotes exon skipping^38,44,47,49^.

We also conditionally ablated hnRNP A1 and hnRNP A2B1 knockout mice by deleting the floxed *Hnrnpa1* and *Hnrnpa2b1* alleles using Mb1-Cre recombinase to study their physiological functions. As functional redundancy might exist due to the high homology between hnRNP A1 and hnRNP A2B1 proteins, we further generated conditional double knockout (DKO) mice with both *Hnrnpa1* and *Hnrnpa2b1* genes deleted specifically in B cells. Immunoblotting assay confirmed that the hnRNP A1 or hnRNP A2B1 proteins were absent in splenic B cells from the individual single and DKO mice (Fig. 8d). Analysis of *Cd40* AS showed that the deletion of either *Hnrnpa1* or *Hnrnpa2b1* could only marginally inhibit exon 6 skipping, as evidenced by the slightly reduced *Cd40* V2 transcript in the single KO compared to the WT B cells (Fig. 8e, lanes 2 and 3). In contrast, the DKO B cells manifested a significant reduction in *Cd40* V2 transcript (Fig. 8e, lane 4). We hypothesized that the decrease in exon 6 skipping would, in turn, increase the *Cd40* V1 transcript leading to an enhanced cell surface expression of CD40. Indeed, we detected a significantly increased CD40 expression on the cell surface of the DKO B cells (Fig. 8f, g).

The opposite phenotypes of CD40 cell surface expression observed in B cells deficient in hnRNP F or A1/A2B1 prompted us to delve into how *Cd40* AS were regulated by these closed but different hnRNP family members. Given that both hnRNP F and A1/A2B1 bind *Cd40* pre-mRNA near its exon 6 region, preferably at the G-tracts, we asked if they compete for the same or overlapping binding sites and exert opposing roles. We first analyzed the *Cd40* pre-mRNA sequences around the exon 6 region to identify the putative hnRNP F- and A1/A2B1-binding sites using the RBPmap (https://rbpmap.technion.ac.il/index.html). We noticed that hnRNP F-binding sites in region 2 of intron 6 overlapped with A1/A2B1-binding sites (Fig. 8h). To prove further that hnRNP F and A1/A2B1 bind *Cd40* pre-mRNA at these overlapping sites, we performed an RNA pull-down assay using biotin-labeled WT and mutant RNAs, with the RNA of reverse sequence (I6-R-biotin) serving as the negative control. One of the mutants was I6-M1M2-biotin RNA, which was used to study hnRNP F’s binding previously (Fig. 7e). We also synthesized another mutant, I6-CM-biotin RNA, in which G within the WT binding region was mutated into C (Fig. 8i). This was necessary because the predicted RNA binding motifs for hnRNP F and A2/A2B1 were slightly different, with the latter being G/A-riched sequences^38^, and therefore G to A mutations might not be able to abolish the binding. The RNA pull-down assays showed that both hnRNP F/H and A1/A2B1 could bind to I6-WT RNA but not the negative control I6-R-biotin (Fig. 8j, lanes 2 and 5). Interestingly, the binding of hnRNP F/H, but not A1/A2B1, was significantly reduced when we used mutant RNA I6-M1M2-biotin, in which G was changed to A (Fig. 8k, lane 3). When we used mutant RNA I6-CM-biotin, in which the binding motifs for both hnRNP F and A1/A2B1 were abolished by G to C mutations, to pull down the proteins, we found that neither hnRNP F/H nor A1/A2B1 could be pulled down (Fig. 8k, lane 4). These data suggest that hnRNP A1/A2B1 and hnRNP F/H bind to the overlapping sites in the region 2 of intron 6 of *Cd40* pre-mRNA and antagonistically regulate *Cd40* AS, with hnRNP A1/A2B1 promoting its exon 6-skipping and thus downmodulating CD40 cell surface expression.

## Discussion

An early study showed that hnRNP F could suppress secretory immunoglobulin expression but favour membrane-bound BCR expression in mouse B cell lines by regulating APA of IgH mRNA^14^. However, our study found a normal expression of membrane-bound BCR in hnRNP F-deficient B cells in mice. This discrepancy could be due to different regulatory mechanisms existing in native B cells versus transformed B cell lines. Additionally, hnRNP H, which also regulate APA of IgH mRNA^14^, could potentially compensate for hnRNP F’s role for APA in the mutant B cells. While B cell development was also largely normal in hnRNP F-deficient mice, the TD antibody response was profoundly compromised due to the lack of hnRNP F in B cells. The mutant mice failed to produce IgH class-switched high-affinity antibodies in response to TD antigen challenge. Concomitantly, hnRNP F-deficient mice had severely compromised GC B cell formation. Our mechanistic studies demonstrated that the mutant B cells exhibited an abnormal AS of *Cd40* mRNA leading to a significantly decreased signaling-competent CD40 expression on the cell surface of B cells. This was supported by the defective proliferation and c-Myc induction in the mutant B cells. Moreover, only the signaling-competent V1 variant, but not the incompetent V2 variant, could partially rescue the CD40 expression and GC B cell defects. However, not all the GC B cells in the chimera mice were from the *Hnrnpf*-deleted B cells rescued with V1. We hypothesize that these rescued cells could not compete with the residual V1-transfected hnRNP F deletion-escaped B cells for expansion, or some other CD40-independent mechanisms may exist downstream of hnRNP F accounting for the incomplete rescue by V1. The defective IgG1 class switching of hnRNP F-deficient B cells induced by LPS and IL-4 also implies that hnRNP F possibly regulates IgG1 class switching beyond controlling CD40 expression. Further investigations are needed to determine if hnRNP F could control GC B cell response by additional mechanisms.

Five *Cd40* mRNA transcript variants can be generated in mouse B cells through AS^42^. Amongst the five variants, only variant 1 encodes a full-length signaling-competent membrane-bound CD40. In contrast, variant 2 has membrane domain-encoding exon 6 skipped, resulting in a truncated secretory form of CD40. The other three variants are also incompetent in transducing CD40 signals, as they either lack intracellular signaling domains of CD40 (variants 3 and 4) or the transmembrane domain (variant 5)^42^. Thus, the aberrant AS of *Cd40* mRNA would lead to impaired CD40 signalling. A recent study reported that CELF1 could suppress *Cd40* exon 6 skipping in human B lymphoma cell line Daudi^27^. Here we showed that hnRNP F could regulate AS of *Cd40* mRNA in mouse B cells and thus control TD antibody response in vivo. These findings highlight the importance of hnRNP F in controlling AS of *Cd40* pre-mRNA in B cells under physiological settings.

Despite the normal B cell development phenotype of hnRNP F-deficient mice, we can not rule out the possibility that hnRNP F plays a role in B cell development. Firstly, CD19-Cre-mediated deletion of floxed *Hnrnpf* alleles was inefficient in the Fr. B to D subsets (Supplementary Fig. 1d), making it difficult to determine hnRNP F’s function in early B development using *Hnrnpf* bKO mice. Secondly, hnRNP H, the homologous proteins of hnRNP F, could compensate for hnRNP F’s deficiency in B cell lineage, making it challenging to explicitly elucidate hnRNP F’s role in early B cell development. HnRNP H1 and hnRNP H2 are 96% identical at the amino acid level, and the amino acid identicalness between hnRNP F and hnRNP H1 is 68%, with that in the third q-RRM being up to 80%^50^.In fact, homologous hnRNP members commonly exist in the same cells and exert overlapping functions. For example, PTBP2 and PTBP3 were upregulated and compensated for the PTBP1 deficit in B cell development in PTBP1-deficient mice^51^. However, PTBP1 conditional knockout mice exhibited a pronounced defect in the positive selection of GC B cells, and PTBP2 could only partially offset the loss of PTBP1. Similarly, our hnRNP F conditional knockout mice also showed very severe defects in GC B cell formation and antibody response, and apparently, the hnRNP H proteins could not compensate for the hnRNP F’s deficiency. These findings indicate that individual hnRNP proteins might play more dominant roles in certain stages or context.

HnRNP proteins primarily regulate AS by binding to a sequence near the target exon^38^. Consistent with this, we found that hnRNP F was also bound to G-tracts adjacent to the 5’ splicing site of the intron 6 of *Cd40* pre-mRNA to promote the exon 6 inclusion. It was reported previously that hnRNP F regulated AS by binding to pre-mRNA G-quadruplex (G4) structures^37^. Therefore, it will be interesting to identify the putative G4 structures within the whole *Cd40* pre-mRNA fragment using the G4 analysis tool QGRS Mapper^52^, and to determine if these G4 structures are necessary for hnRNP F-mediated regulation of *Cd40* AS. Earlier studies also demonstrated that hnRNP F could recruit components of splicing factors U1 or U2 to promote the target exon splicing^12,13^. We speculate that hnRNP F might promote intron 6 removal and exon 6 inclusion by recruiting the U1 splicing factors because hnRNP F could bind to the region adjacent to the 5’ splicing site of intron 6. Furthermore, G-tracts were not found within exon 6 and at the 3’ splicing site of intron 6. The same model might also apply to explain the removal of *Cd40* intron 7, as hnRNP F could bind to the G-tracts adjacent to its 5’ splicing site as well.

Another interesting finding from our study was that hnRNP A1 and A2B1 could suppress *Cd40* exon 6 skipping. It is known that AS are mainly regulated by the hnRNP protein complexes, and some combinations of hnRNPs act synergistically while others exert antagonistic functions. Earlier studies have shown that hnRNP A1 and hnRNP F can regulate AS of the same pre-mRNA but oppositely^38,44^. Here we showed that hnRNP A1/A2B1 and hnRNP F also had opposing roles in regulating the AS of *Cd40*, with hnRNP A1/A2B1 suppressing and hnRNP F promoting exon 6 inclusion. Interestingly, both hnRNP A1/A2B1 and hnRNP F have been found to bind to G-tracts for AS regulation^9,38^, suggesting that hnRNP A1 and A2B1 may compete with hnRNP F for the same binding site on *Cd40* pre-mRNA. Without the competition of hnRNP F, as in the case of hnRNP F-deficient B cells, hnRNP A1 and A2B1 could bind even more readily to the 5’ splice site of *Cd40* pre-mRNA intron 6, thus promoting exon 6 skipping. Therefore, the availability of different *Cd40* variants in B cells is determined by the balanced effect of the competitive and co-regulatory hnRNP proteins. Future study of hnRNP A1/A2B1 DKO mice and mice deficient in all three hnRNPs might shed more light on how these molecules regulate *Cd40* AS in B cells.

In summary, our study showed that hnRNP F is required to produce high affinity IgH class-switched antibodies during TD antibody response and promote GC B cell formation. Mechanistically, hnRNP F controlled the appropriate cell surface expression of CD40 in B cells by regulating the AS of *Cd40* pre-mRNA. Furthermore, we discovered that hnRNP A1 and A2B1 are also involved in regulating AS of *Cd40* but have an opposite effect to hnRNP F, suggesting they work in concert to regulate AS of *Cd40* to ensure optimal GC B cell formation and antibody response.

## Methods

### Mice and immunization

All experimental animal operations were approved by the Institutional Animal Care and Use Committee of the Southern University of Science and Technology. *Cd19*^Cre/+^ (B6.129P2(C)-*Cd19*^*tm1(cre)Cgn*^/J, strain #: 007770) and *Mb1*^Cre/+^ (B6.C(Cg)-*Cd79a*^*tm1(cre)Reth*^/EhobJ, strain #: 020505) mice were obtained from The Jackson Laboratory. *Hnrnpf* floxed mice were obtained from Nanjing Biomedical Research Institute of Nanjing University (Cat #: T000629). To generate conditional knockout mice, the *Hnrnpf* floxed mice were crossed to Flp expressing mice (Cyagen, Cat #: C001090) to remove the neo-cassette and then crossed to *Cd19*^Cre/+^ mice to delete exon 4 of *Hnrnpf* gene (Supplementary Fig. 6a). *Hnrnpa2b1* floxed mice (Cat #: S-CKO-11602) were obtained from Cyagen and crossed with *Mb1*^Cre/+^ to generate conditional knockout mice to delete its exons 2 to 6 (Supplementary Fig. 6b). *Hnrnpa1* floxed mice (Cat #: T013507) were obtained from Gempharmatech and crossed with *Mb1*^Cre/+^ to generate conditional knockout mice to delete its exons 2 to 11 (Supplementary Fig. 6c). Male and female mice of 6–12 weeks old were used for breeding and experiments. For chimera assay, muMT (B6.129S2-*Ighm*^*tm1Cgn*^/J, strain #: 002288) mice were obtained from The Jackson Laboratory and the recipients were wild-type C57BL/6 J mice (The Jackson Laboratory, strain #: 000664). All mice were on a C57BL/6 background and feeded with SPF grade Rats & Mice Growing and Breeding feed (Beijing Keao Xieli Feed Co.,Ltd., Cat. 1016706476803973120). All mice were housed under specific pathogen-free conditions and on a 12 h on/12 h off light/dark cycle with temperature 20–26 °C and humidity ~70% in the Laboratory Animal Center of the Southern University of Science and Technology. For experiments, the mice were euthanized in a CO_2_ chamber.

For T-cell dependent antibody immune response, mice were immunized with 100 μg NP_30_-CGG or NP_30_-KLH (Biosearch Technologies) in alum (Thermo) intraperitoneally.

### Cell culture and B cell stimulation in vitro

3T3-L1 fibroblast cells were a gift from the Life Science Experimental Teaching Demonstration Center of Southern University of Science and Technology, and were cultured with DMEM high glucose liquid media (Hyclone) containing 10% fetal bovine serum (ExCell Bio), 100 U/ml penicillin and 100 μg/ml streptomycin (Hyclone) in 24-well plates or 100 mm dishes (NEST Biotechnology) at 37°C with 5% (v/v) CO2. HEK 293 T cells were cultured under the same conditions as 3T3-L1 fibroblast cells.

For B cell stimulation in vitro, anti-CD43 beads (Miltenyi Biotec) and LS columns (Miltenyi Biotec) were used for the purification of splenic B cells. Two million purified splenic B cells were cultured in RPMI-1640 medium (Gibco) supplemented with 10% fetal bovine serum (Gibco), 100 U/ml penicillin and 100 μg/ml streptomycin (Hyclone). LPS (20 µg/ml, Sigma) was used to stimulate B cells for retroviral transduction. Anti-CD40 (1 µg/ml, BD Biosciences) antibody was used for immunoblot analysis of NF-κB pathway in splenic B cells upon CD40 engagement.

### CSR and cell proliferation in vitro

Mouse splenic B cells were isolated using EasySep™ Mouse B Cell Isolation Kit (Stem Cell) and stimulated with 20 μg/ml LPS (Sigma-Aldrich) for CSR to IgG3. For CSR to IgG1, B cells were stimulated with 20 μg/ml LPS (Sigma-Aldrich) and 10 ng/ml of mouse recombinant IL-4 (Bio-Techne, # 404-ML), or 1 μg/ml anti-CD40 (BD Biosciences) and 10 ng/ml of mouse recombinant IL-4 (Bio-Techne, # 404-ML), or 1 μg/ml anti-CD40 (BD Biosciences), 10 ng/ml of mouse recombinant IL-4 (Bio-Techne, # 404-ML) and 10 ng/ml of mouse recombinant IL-21 (Bio-Techne, # 594-ML).

For the proliferation assay, cells were stained with CellTrace™ Far Red Cell Proliferation Kit (Thermofisher) for 5 min at 37 °C and cultured with indicated stimulation. Cells were analyzed at day 0 and day 3 using flow cytometry.

### iGB cell culture

Splenic B cells were purified using EasySep™ Mouse B Cell Isolation Kit (Stem Cell) and seeded with 10^6^ cells per well of 96-well plate in 200 ul RPMI-1640 medium (Gibico) supplemented with 15 % FBS (ExCell Bio), 55 μM 2-mercaptoethanol (Sigma), 1 X Non-Essential Amino Acids (Gbico), 10 mM HEPES (Gibico), 1 mM sodium pyruvate, 100 U/ml penicillin and 100 μg/ml streptomycin (Hyclone). B cells were stimulated with 1 μg/ml of anti-CD40 (BD Biosciences), 5 ng/ml of mouse recombinant IL-4 (Bio-Techne, # 404-ML), and 5 ng/ml of mouse recombinant IL-21 (Bio-Techne, # 594-ML) for primary culture. After 1 day, Cells from each well of 96-well plate were transferred to 10 cm plates containing 10 ml medium and then cultured with the same stimulation for another 3 days. iGB cells were analyzed at day 4 using flow cytometry.

### Plasmid construction

All plasmids used in this study were constructed in a MIGR1-based vector as described previously^53^. For the construction of MIGR1-CD40-V1, MIGR1-CD40-V2, MIGR1-CD40-V6 and MIGR1-Hnrnpa1, target DNAs were amplified using KOD-Plus-neo (TOYOBO) from cDNA library of mouse splenic B cells and then inserted into the MIGR1 vector using ClonExpress MultiS one Step Cloning Kit (Vazyme). For MIGR1-CD40-minigene construction, we used a minigene composed of mouse CD40 coding sequence and introns 5 (excluding the region spanning 5′-CATGTGCATGCATGC…ATCTGATCATCTTTT-3′), 6 (excluding the regions spanning 5′- CAGTGTCACCTGGTA…GCACAGATGCACACA-3′), 7 and 8 (Fig. 6b). Sanger sequencing was used for the confirmation of all inserted sequences.

### Transfection and retrovirus transduction

3T3-L1 cells were transiently transfected with WT or mutant MIGR1-CD40-minigene plasmids coated by Liposome Transfection Reagent (Yeasen) in OPTI-MEM (Gibco), cultured for 48 h, and then collected for following experiments. For the transfection in 24-well plates, 8 × 10^4^ 3T3-L1 cells were seeded, and 1 μg of plasmids mixed with 2 uL of transfection reagent was added for each well. HEK 293 T cells were transiently transfected with MIGR1-CD40-V1, -V2 or/-V6 using the same reagents as 3T3-L1 cells.

For retrovirus transduction, HEK 293 T cells were co-transfected with equal amounts of MIGR1 or MIGR1-based plasmids and pCL-ECO (retrovirus packaging vector) plasmids using Lipofectamine™ 3000 Transfection Reagent (Thermo). The virus supernatant was collected at 48 h and 72 h after transfection and added to target cells in a fresh culture medium with 6 μg/ml polybrene (Sigma) and cytokines or stimulators. Then target cells were centrifuged at 600 × *g* at 37 °C for 1.5 h. After two rounds of transductions, cells were cultured for a further 24 h or 48 h followed by the subsequent analysis or experiments.

### Flow cytometry

Single-cell suspensions were prepared from mouse spleens or BM by removal of red blood cells using RBC lysis buffer (BioLegend) and passing through 70-µm nylon mesh. Cells were then stained with indicated antibodies in PBS containing 5% (v/v) FBS at 4 °C. NP-specific antibodies were detected by NIP-BSA (Biosearch Technologies) conjugated with PE as described previously^54^. Dead cells were excluded using DAPI staining. The data were collected using CytoFLEX S flow cytometry analyzer with CytExpert software (Beckman Coulter, V2.3). The geometric mean fluorescence intensity was calculated by Flowjo V10.8.1.

For FOB cell sorting, single-cell suspensions were prepared and stained as described above and the cells (B220^+^CD93^−^CD23^high^CD21^low^) were sorted on a BD FACSAiraII (BD bioscience).

### ELISPOT assay

ELISPOT assay was performed as described previously^55^. In brief, MultiScreen filter plates with 0.45 μm PVDF membrane (Milipore) were coated with 1 μg per well NP_20_-BSA (Biosearch Technologies) in PBS overnight at 4°C and blocked with 2% BSA in PBS for 2 h at room temperature. One million splenocytes or three million BM cells were seeded in each well, and then the plate was incubated at 37°C for 2 h. After washing, the plate was incubated with Biotinylated anti-IgM (1: 1000, SouthernBiotech, 1140-08) or anti-IgG1 (1: 1000, SouthernBiotech, 1144-08) antibodies for 1 h at room temperature, followed by streptavidin-AP (1:1000, SouthernBiotech, 7105-04) incubation for 0.5 h at room temperature. The substrates (MABTECH) were added to each well for visualization of antibody-secreting cells.

### ELISA

NP-specific antibodies levels were detected using ELISA as described previously^56^. Briefly, sera were collected before and after NP-CGG immunization on the indicated days. NP_20_-BSA or NP_2_-BSA (Biosearch Technologies) were used for capturing NP-specific antibodies and total immunoglobulins were captured by Goat Anti-Mouse Immunoglobulins (SouthernBiotech, 1010-01). HRP-conjugated anti-IgG1 (1:1000, SouthernBiotech, 1071-05), -IgG2b (1:1000, SouthernBiotech, 1091-05), -IgG3 (1:1000, SouthernBiotech, 1101-05), -IgA (1:1000, SouthernBiotech, 1040-05) and -IgM (1:1000, SouthernBiotech, 1021-05) antibodies conjugated with HRP were used as secondary antibodies, followed by TMB substrate (BioLegend) development. OD450 values were measured using a microplate reader with Gene5 Software (BioTek, V2.08).

### RNA isolation, RT-PCR and qRT-PCR

RT-PCR was performed as described previously^42^. Briefly, total RNA was purified using RNAiso Plus (Takara) from measured cells. cDNA was reversely synthesized with RT SuperMix (Vazyme) and amplified with appropriate primers using 2× Taq Master Mix (Vazyme), and the PCR products were analyzed through agarose gel electrophoresis. Image J (V1.53e) was used for RT-PCR gel gray scale analysis. RT-PCR primers used for AS of *Cd40* are as follows: P1, 5′-CTGCCCAGTCGGCTTCTTCTC-3′; P2, 5′-CCTGTGTGACAGGCTGACAC-3′; P3, 5′-GTTTAAAGTCCCGGATGCGA-3′; P4, 5′-CTCATTATCCTTTGGTTTCTTGACC-3′. Primers for semiquantitative analysis: *Actin* forward, 5′-CGTGAAAAGATGACCCAGATCA-3′; *Actin* reverse, 5′-CACAGCCTGGATGGCTACGT-3′; *Cd40* forward, 5′-GCAGTGTGTTACGTGCAGTG-3′; *Cd40* reverse, 5′-TGTGCAGTGGCTTGTCAGTC-3′; *Hnrnpa1* forward, 5′-AACCGACGAGAGTCTGAGGA-3′; *Hnrnpa1* reverse, 5′-CAAACCCAAAGCCCCTGGAT-3′.

For qRT-PCR, RNA extraction and cDNA synthesis were performed according to the above mentioned methods. Quantitative real-time PCR was performed with ChamQ Universal SYBR qPCR Master Mix (Vazyme). The primer sequences for qRT-PCR used in this study were as follows: *Myc* forward, 5′-GTGCTGCATGAGGAGACACC-3′, *Myc* reverse, 5′-GACCTCTTGGCAGGGGTTTG-3′; *Hnrnpf* forward, 5′- AGAAGGCATCTGTGGTGGTT-3′, *Hnrnpf* reverse, 5′- GAAATGAACACCTGCGACCC −3′.

### Immunoblotting

Immunoblotting was performed as described previously^57^. Briefly, whole-cell lysates were prepared using RIPA buffer (Sigma-Aldrich) containing protease inhibitor cocktail (Roche). Proteins were separated by running on 10% SDS-PAGE gels and transferred to PVDF membranes. After blocking with 5% skim milk in TBST, membranes were probed with appropriate primary antibodies overnight and further incubated with goat anti-rabbit IgG-HRP (Proteintech). Blots were developed using Western chemiluminescent HRP substrate (Millipore) and visualized using an imaging system (Tanon, 5200). The primary antibodies used in immunoblotting are as follows: β-actin (1:2000, Proteintech, 20536-1-AP), hnRNP F (1:1000, Thermo, PA522341), hnRNPA1 (1:1000, Cell Signaling Technology, #8443), hnRNPA2B1 (1:1000, ABclonal, A1162), p-IKKα/IKKβ (1:1000, Cell Signaling Technology, #2078), IKKβ (1:1000, Cell Signaling Technology, #8943), p-IKBα (1:1000, Cell Signaling Technology, #2859) and IKBα (1:1000, Cell Signaling Technology, #9242).

### Chimera assay

Chimera assay was performed as described previously^58^. In brief, donor mice were intraperitoneally injected with 0.15 mg/g 5-FU (Sigma) according to their body weight for 3 days. BM cells were then collected and cultured in a fresh medium containing 20 ng/ml of IL-3 (Bio-Techne, # 403-ML), 50 ng/ml of IL-6 (Bio-Techne, # 406-ML) and 50 ng/ml of SCF (Bio-Techne, # 455-MC). After 24 h, cells were collected with a cell scraper (NEST Biotechnology) and transduced by retrovirus using the method described above. The cells were then injected intravenously into irradiated recipient mice (900 rads) to generate chimera mice.

### RIP-qPCR

Native RNA immunoprecipitation (RIP) was performed as described previously^59^. Briefly, mouse splenic B cells were lysed and centrifuged to isolate the supernatant cell lysate. After taking 10 % of the supernatant as input, the cell lysate (100% to input) was incubated with immobilized hnRNP F antibody (Thermo, PA522341) to Protein G Magnetic Beads (Cell Signaling Technology, #9006) overnight at 4 °C. After extensive washing, immunoprecipitation complexes were digested using Proteinase K (Beyotime) and processed for RNA purification using RNAiso Plus (Takara). IP and input RNAs were reversely transcribed with RT SuperMix (Vazyme) to produce cDNA. Quantitative real-time PCR was performed with ChamQ Universal SYBR qPCR Master Mix (Vazyme) using primers CD40-E6-F (5′-CTGTGAGGATAAGAACTTGGAG-3′) and CD40-E6-R (5′-CACAGATGACATTAGTCTGACT-3′) to amplify *Cd40* RNA. Fold enrichment was calculated as described previously^59^.

### RNA pull-down and mass spectrometry

Biotin-labelled RNAs were synthesized by GENEWIZ or transcribed using T7 RNA polymerase (Beyotime) combined with Biotin RNA Labeling Mix (Sigma) in vitro. RNA pull-down assay was performed according to a previous report with some modifications^37^. Two μg biotinylated RNA in vitro or 250 pmol biotinylated RNA oligos were immobilized to 30 μl Streptavidin Magnetic Beads (MedChemExpress) according to the manufacturer’s instruction. Then RNA-beads complexes were incubated with 100 μg nuclear extracts from mouse splenic B cells in 250 μl pull-down buffer (20 mM Tris-HCl pH = 7.5, 200 mM NaCl, 6 mM EDTA, 5 mM potassium fluoride, 5 mM β-Glycerophosphate, 2 mg/mL protease inhibitor cocktail) for 4 h at 4 °C. The beads were washed five times with pull-down buffer and then dissolved in western blot loading buffer for loading onto 10% SDS-PAGE gels.

For immunoblotting, proteins in gels were transferred to PVDF membranes for further detection. For protein mass spectrometry analysis, proteins in gels were sliced for label-free protein quantification. MaxQuant software (version 1.6.10.43) was used for MS/MS analysis using *Mus musculus* database (55470 sequences) with the followed parameters: oxidation of methionine protein N-terminal acetylation, variable modifications; carbamidomethylation, fixed modifications; digestion enzyme, trypsin/P; a maximum of peptide charge, 6 and 2 missed cleavages/peptide allowed; mass tolerance for precursor ions, 20 p.p.m for the first search and 7 p.p.m for the main search; FDRs of peptide and protein, 1%.

### EMSA

The mouse recombinant hnRNP F and hnRNP H1 proteins were purified as previously described^37^. In brief, hnRNP F and H1 proteins were expressed in *E. coli* Rosetta induced by 0.5 mM IPTG, and purified from supernatant using BeaverBeads™ His-tag Protein Purification beads (Beaver) after sonication. Binding of *Cd40* RNA and hnRNP F/H was detected using the BersinBio^TM^ RNA-EMSA Kit (BersinBio) according to the operating manual. For each reaction, 12.5 nM biotin-labeled RNAs were incubated with 200 ng purified protein.

### RNA-seq and data processing

Total RNAs were exacted from FACS-sorted FOB cells from WT and *Hnrnpf* KO mice using RNeasy Mini Kit (Qiagen). After quantification and poly(A) mRNA isolation, the mRNA was fragmented and subjected to library preparation using NEBNext Ultra RNA Library Prep Kit for Illumina. The library DNA was loaded on an Illumina NovaSeq 6000 instrument 150 bp paired-end (PE150) configuration carried out by GENEWIZ. The software FastQC (v0.10.1) was used to detect the sequencing quality of the raw data, and Cutadapt (v1.9.1) was used to filter the raw data to get clean reads. Clean reads were aligned to GRCm38 (Ensembl) using Hisat2 (v2.0.1), and gene expression levels were estimated by HTSeq (v0.6.1). Genes with differential expression were obtained using DESeq2 (v1.6.3) Bioconductor package. KEGG analysis of differentially expressed (fold change ≥ 2, adjusted *p* ≤ 0 .05) genes was performed using R (v3.6.3) package ClusterProfiler (v3.14.3). rMATS turbo (v4.1.0) was used for analyzing differential AS events with mapped BAM files from above and with parameter: -variable-read-length.

### Sequencing data visualization and statistical analysis

The volcano plot of sequencing data was obtained using the OmicStudio tools at https://www.omicstudio.cn/tool. Sashimi plot of differential exons junction in WT or *Hnrnpf* KO B cells was generated using the IGV (http://www.igv.org/). Statistical analysis was performed using GraphPad Prism 7, and the significance tests we used were indicated in the figure legends. *P* value <0.05 was considered as the threshold for statistical significance.

### Reporting summary

Further information on research design is available in the Nature Portfolio Reporting Summary linked to this article.

## Supplementary information

Supplementary Information

Description of Additional Supplementary Files

Supplementary Data 1

Supplementary Data 2

Reporting Summary

## Data Availability

RNA-Seq data of *Hnrnpf* expression in B cell subsets were obtained from the Immunological Genome Project (ImmGen) database (https://www.immgen.org/). RNA-Seq data (including raw data, gene expression profiling, and changed alternative splicing events) produced in this study have been deposited in the GEO database (https://www.ncbi.nlm.nih.gov/geo/query/acc.cgi?acc=GSE188538) with the accession number GSE188538. Data for LC/MS/MS of CD40 mRNA pull-down have been deposited to Figshare (10.6084/m9.figshare.19750645.v1). Source data are provided with this paper.

## Source data

Source Data
