# Supplementary material for: The RNA-binding protein hnRNP F is required for the germinal center B cell response
Source: Nat Commun. 2023 Mar 30;14:1731. doi: 10.1038/s41467-023-37308-z (PMC10063658; doi:10.1038/s41467-023-37308-z)
Supplement: Supplementary file 1 — Supplementary Information [file 41467_2023_37308_MOESM1_ESM.pdf]

Supplementary Information

The RNA-binding protein hnRNP F is required for the germinal center B cell response

Hengjun Huang, Yuxing Li, Gaopu Zhang, Gui-Xin Ruan, Zhijian Zhu, Wenjing Chen, Jia Zou, Rui Zhang, Jing Wang, Yu Ouyang, Shengli Xu, and Xijun Ou

Supplementary Figure 1

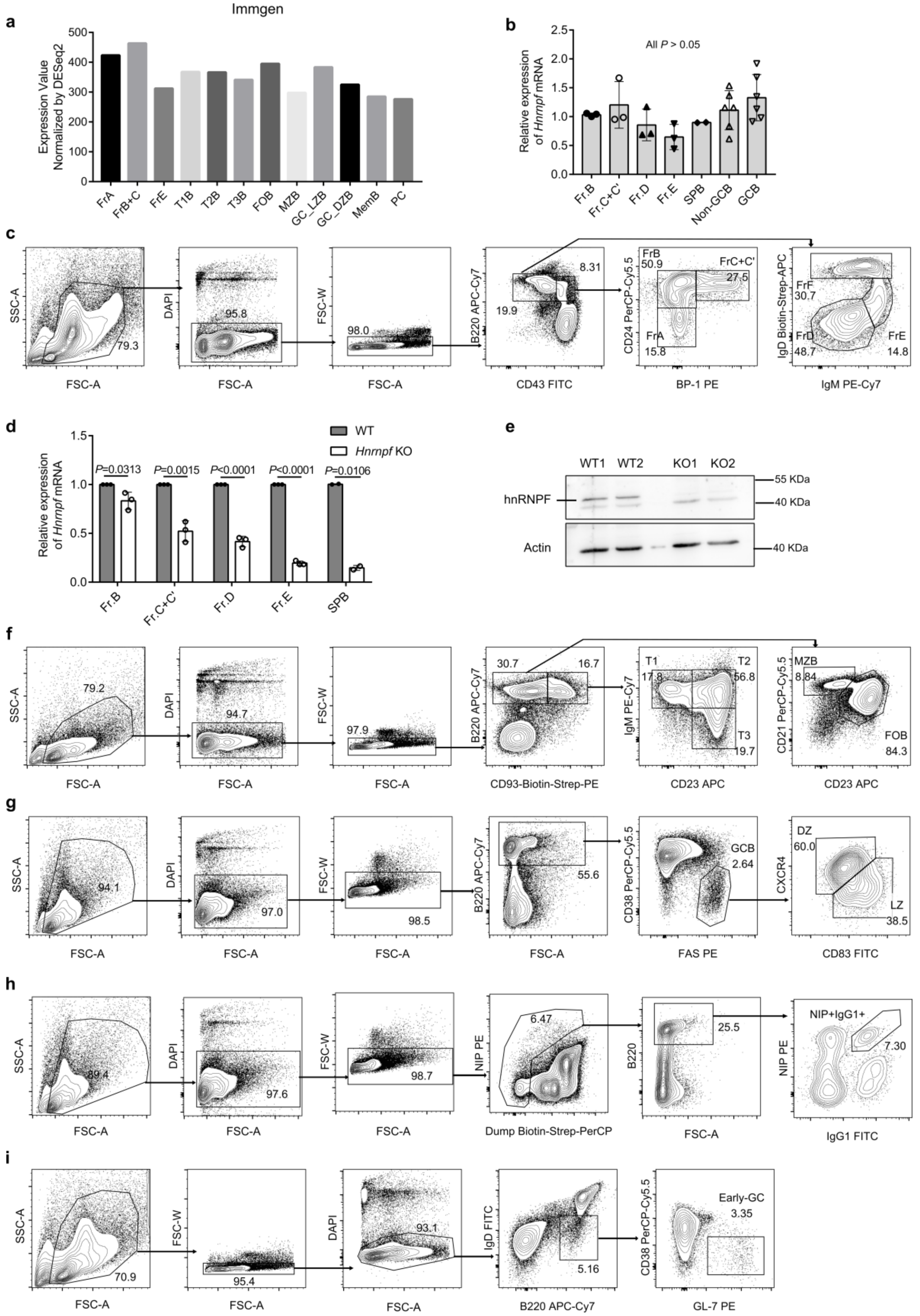

Supplementary Figure 1.The *Hnrnpf* expression level in B cell subsets and the gating history of indicated B cell populations.

a The *Hnrnpf* expression in various mouse B cell subsets (data from the Immunological Genome Project RNA-Seq datasets, GSE109125). **b** qRT-PCR analysis of *Hnrnpf* expression in different subsets of mouse B cells (n = 2 to 6). One-way ANOVA without adjustment was used for the statistical analysis. **c** Gating strategy for analyzing B cell development in BM, related to Fig.1a. **d** qRT-PCR analysis of *Hnrnpf* deletion efficiency in different B cell subsets of *Hnrnpf* KO mice (n = 3 per group). An unpaired two-tailed student's t test was used for the statistical analysis. **e** Western blot analysis of *Hnrnpf* expression in total splenic B cells of WT and *Hnrnpf* KO mice. Data are representative of two independent experiments. **f** Gating strategy for analyzing B cell development in spleen, related to Fig. 1c. **g**, **h** Gating strategies for analyzing GC B cells (**g**) and antigen-specific B cells (**h**) in the spleen at day 10 post-immunization, related to Fig. 4a and 4c. **i** Gating strategy for analyzing early GC B cells in the spleen at day 5 post-immunization, related to Fig. 4e. Each dot represents an individual mouse, and data are pooled from two independent experiments (**b** and **d**). Data are presented as mean values ± SD (**b** and **d**). Source data are provided as a Source Data file.

Supplementary Figure 2

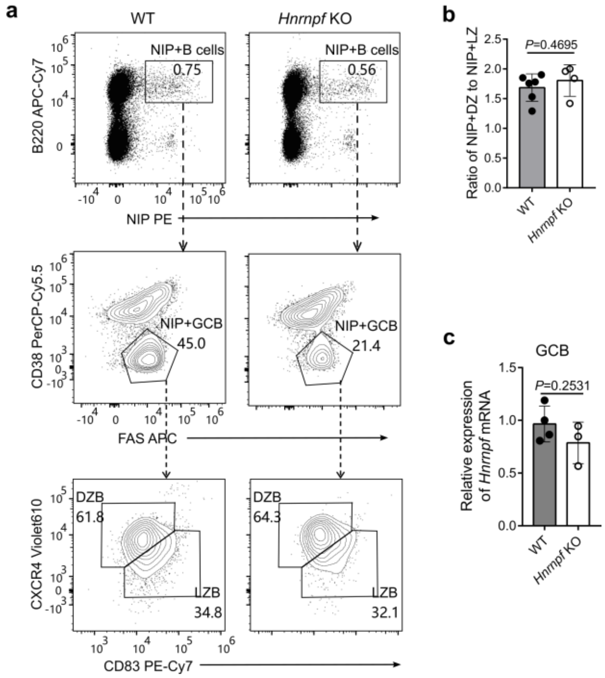

Supplementary Figure 2. Expansion of residual hnRNP F-sufficient GC B cells in *Hnrnpf* KO mice.

a, **b** Flow cytometric analysis of NP specific DZ and LZ B cells in the spleen of WT and *Hnrnpf* KO mice at day 10 post-immunization (n = 6 for WT group and n = 4 for *Hnrnpf* KO group). **c** qRT-PCR analysis of *Hnrnpf* expression in GC B cells from WT and *Hnrnpf* KO mice at day 10 post-immunization (n = 4 for WT group and n = 3 for *Hnrnpf* KO group). Data are representative of four independent experiments (**a**) or three independent experiments (**c**). An unpaired two-tailed student's t test was used for the statistical analysis (**b** and **c**). Data are presented as mean values ± SD (**b** and **c**). Source data are provided as a Source Data file.

Supplementary Figure 3

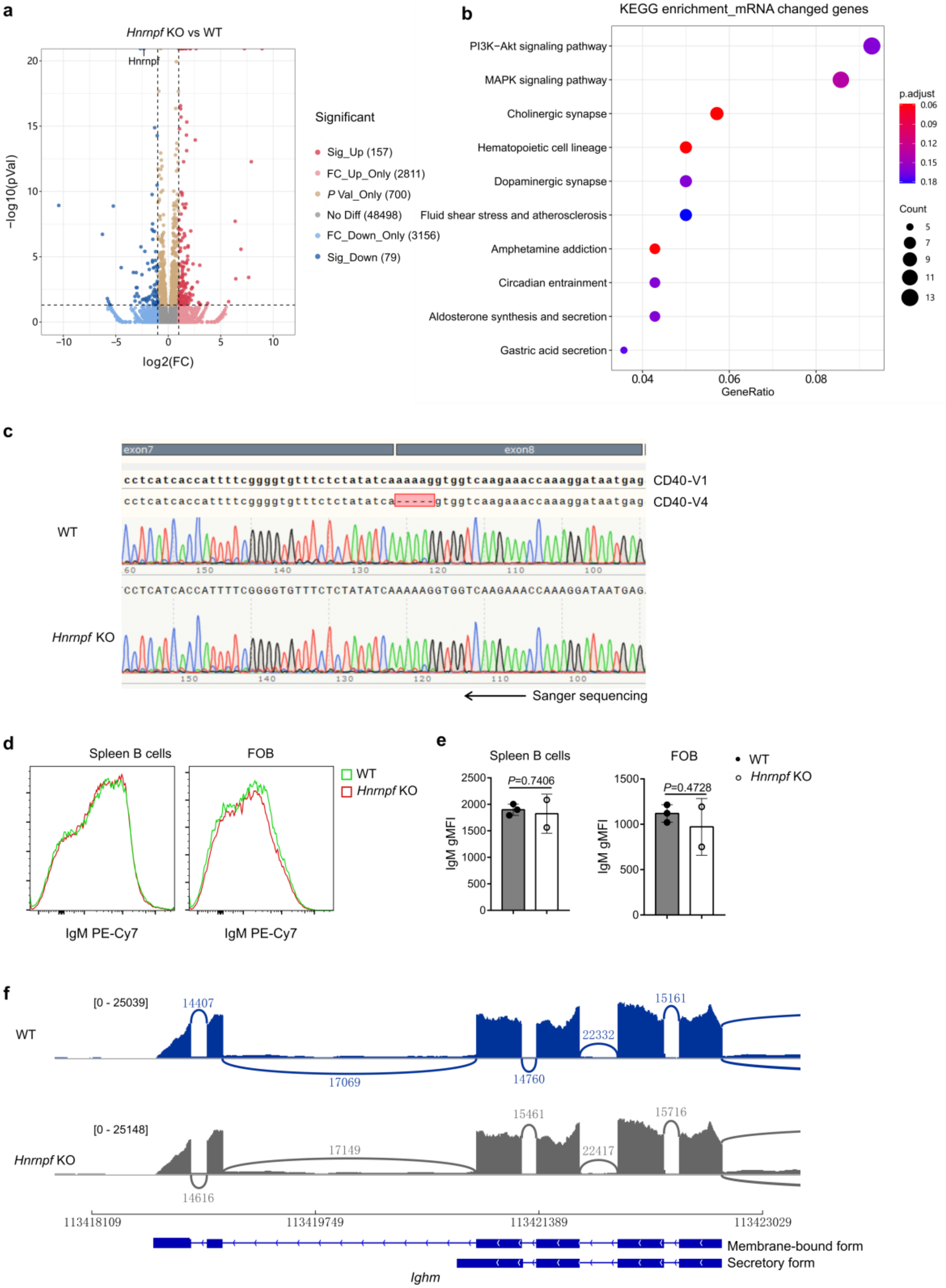

Supplementary Figure 3. RNA-seq and IgM expression analysis of WT and *Hnrnpf* KO B cells.

a The volcano plots of significant differentially expressed genes (|fold change| ≥ 2, FDR ≤ 0.05) in FO B cells of WT and *Hnrnpf* KO mice. The statistical analysis was performed using DESeq2 Bioconductor package based on the negative binomial distribution and Benjamini-Hochberg method. **b** KEGG enrichment analysis of differentially expressed genes based on Hypergeometric test as shown in **a**. **c** CD40-V1 and CD40-V4 were identified by Sanger sequencing using the primer CD40-P2. **d**, **e** Flow cytometry analysis of IgM expression on the cell surface of splenic and follicular B cells (n = 3 for WT group and n = 2 for *Hnrnpf* KO group). An unpaired two-tailed student's t test was used for the statistical analysis. Each symbol represents an individual mouse. **f** Sashimi plot displaying *Ighm* isoform expression in WT and *Hnrnpf* KO follicular B cells. The membrane-bound form and secretory form of *Ighm* were indicated. Data are representative of two independent experiments (**d**). Data are presented as mean values ± SD (**e**). Source data are provided as a Source Data file.

Supplementary Figure 4

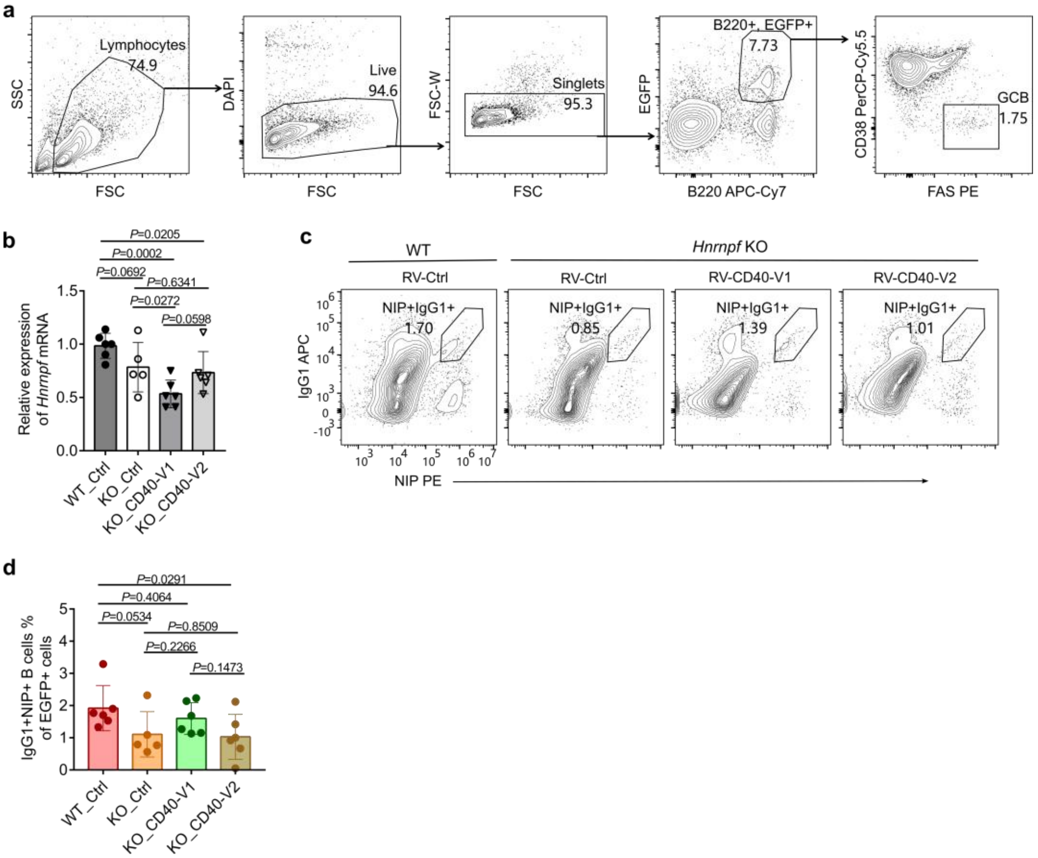

Supplementary Figure 4. hnRNP F promotes GC B cell formation through regulating CD40 AS.

a The gating strategies for analyzing EGFP$^+$ GC B cells in the chimeric mice at day 10 post-immunization, related to Fig. 6i. The EGFP$^+$B220$^+$ cells were retrovirus-infected B cells, representing either empty plasmids or plasmids with CD40 variants. **b** Knockout efficiency of *Hnrnpf* in GC B cells in the chimeric mice at day 10 post-immunization. **c**, **d** Flow cytometric analysis of NP-specific IgG1$^+$ B cells (gated from B220$^+$EGFP$^+$ cells) in the spleen of chimeric mice at day 10 after immunization. Data are pooled from three independent experiments with the mean of n = 6 mice per group (**b**, **d**). One-way ANOVA without adjustment was used for the statistical analysis (**b**, **d**). Data are presented as mean values ± SD (**b**, **d**). Source data are provided as a Source Data file.

Supplementary Figure 5

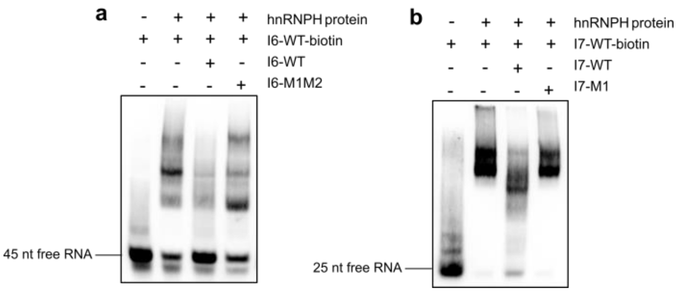

Supplementary Figure 5. hnRNP H binds to *Cd40* pre-mRNA.

a, **b** RNA EMSA detection of hnRNP F binding to intron 6 (**a**) and intron 7 (**b**) of *Cd40* pre-mRNA. Data are representative of two independent experiments. Source data are provided as a Source Data file.

Supplementary Figure 6

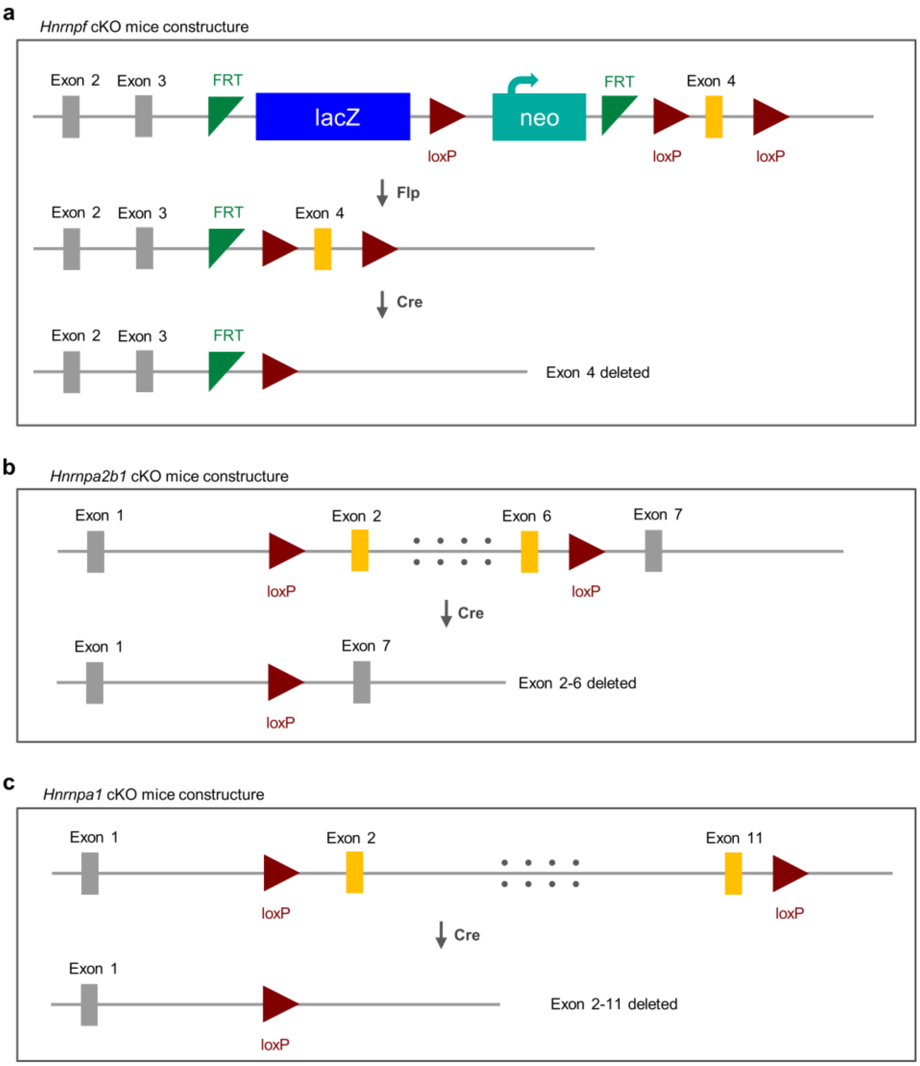

Supplementary Figure 6. Gene targeting strategies for the generation of different conditional knockout mice.

a-c Gene targeting strategies for *Hnrnpf* (**a**), *Hnrnpa2b1* (**b**), and *Hnrnpa1* (**c**) alleles were shown.
