# Supplementary material for: The RNA-binding protein hnRNP F is required for the germinal center B cell response
Source: Nat Commun. 2023 Mar 30;14:1731. doi: 10.1038/s41467-023-37308-z (PMC10063658; doi:10.1038/s41467-023-37308-z)
Supplement: Supplementary file 2 — Description of Additional Supplementary Files [file 41467_2023_37308_MOESM2_ESM.pdf]

Description of Additional Supplementary Files

File Name: Supplementary Data 1

Description: List of genes differentially expressed between WT and *Hnrnpf* bKO follicular B cells.

File Name: Supplementary Data 2

Description: Alternative splicing events identified from the RNA-Seq data of WT and *Hnrnpf* bKO follicular B cells.
