# Supplementary material for: The RNA-binding protein hnRNP F is required for the germinal center B cell response
Source: Nat Commun. 2023 Mar 30;14:1731. doi: 10.1038/s41467-023-37308-z (PMC10063658; doi:10.1038/s41467-023-37308-z)
Supplement: Supplementary file 5 — Reporting Summary [file 41467_2023_37308_MOESM5_ESM.pdf]

nature portfolio

Corresponding author(s): Xijun Ou

Last updated by author(s): Feb 23, 2023

Reporting Summary

Nature Portfolio wishes to improve the reproducibility of the work that we publish. This form provides structure for consistency and transparency in reporting. For further information on Nature Portfolio policies, see our Editorial Policies and the Editorial Policy Checklist.

Statistics

For all statistical analyses, confirm that the following items are present in the figure legend, table legend, main text, or Methods section.

n/a	Confirmed	
☐	☒	The exact sample size (*n*) for each experimental group/condition, given as a discrete number and unit of measurement
☐	☒	A statement on whether measurements were taken from distinct samples or whether the same sample was measured repeatedly
☐	☒	The statistical test(s) used AND whether they are one- or two-sided<br>*Only common tests should be described solely by name; describe more complex techniques in the Methods section.*
☒	☐	A description of all covariates tested
☐	☒	A description of any assumptions or corrections, such as tests of normality and adjustment for multiple comparisons
☐	☒	A full description of the statistical parameters including central tendency (e.g. means) or other basic estimates (e.g. regression coefficient) AND variation (e.g. standard deviation) or associated estimates of uncertainty (e.g. confidence intervals)
☐	☒	For null hypothesis testing, the test statistic (e.g. *F*, *t*, *r*) with confidence intervals, effect sizes, degrees of freedom and *P* value noted<br>*Give P values as exact values whenever suitable.*
☒	☐	For Bayesian analysis, information on the choice of priors and Markov chain Monte Carlo settings
☒	☐	For hierarchical and complex designs, identification of the appropriate level for tests and full reporting of outcomes
☒	☐	Estimates of effect sizes (e.g. Cohen's *d*, Pearson's *r*), indicating how they were calculated

Our web collection on statistics for biologists contains articles on many of the points above.

Software and code

Policy information about availability of computer code

Data collection	Flow cytometry data were collected using CytExpert software (Beckman Coulter, v2.3).<br>OD450 values were collected using Gen5 Software (Bio Tek, v2.08).
Data analysis	Flow cytometry data were analyzed using FlowJo (versions 10.8.1).<br>Statistical analysis was carried out using Prism (versions 7).<br>Image J (V1.53e) was used for RT-PCR gel gray scale analysis.<br>MaxQuant software (version 1.6.10.43) was used for MS/MS analysis.<br>Softwares used for RNA-seq analysis were listed as follows: Cutadapt (V1.9.1), FastQC (V0.10.1), HISAT2 (V2.0.1), HTSEQ (V0.6.1), DESeq2 (V1.6.3), R (V3.6.3), and ClusterProfiler (V3.14.3).<br>rMATS turbo (v4.1.0) was used for analyzing differential AS events.<br>IGV genome browser (V2.7.2) was used for exon junction visualization.<br>The volcano plot was obtained using the OmicStudio tools (VolcanoPlot_V4.0).

For manuscripts utilizing custom algorithms or software that are central to the research but not yet described in published literature, software must be made available to editors and reviewers. We strongly encourage code deposition in a community repository (e.g. GitHub). See the Nature Portfolio guidelines for submitting code & software for further information.

Data

Policy information about availability of data

All manuscripts must include a data availability statement. This statement should provide the following information, where applicable:

- Accession codes, unique identifiers, or web links for publicly available datasets
- A description of any restrictions on data availability
- For clinical datasets or third party data, please ensure that the statement adheres to our policy

Expression data of Hnrnpf during B cell development were obtained from the Immunological Genome Project (ImmGen) database (https://www.immgen.org/).
RNA-Seq data has been deposited in the GEO database with the accession number GSE188538;
Data for LC/MS/MS of CD40 mRNA pull-down have been deposited to Figshare (https://doi.org/10.6084/m9.figshare.19750645.v1)
Source data of gels and blots are provided with this paper.

Human research participants

Policy information about studies involving human research participants and Sex and Gender in Research.

Reporting on sex and gender	n/a
Population characteristics	n/a
Recruitment	n/a
Ethics oversight	n/a

Note that full information on the approval of the study protocol must also be provided in the manuscript.

Field-specific reporting

Please select the one below that is the best fit for your research. If you are not sure, read the appropriate sections before making your selection.

☒ Life sciences ☐ Behavioural & social sciences ☐ Ecological, evolutionary & environmental sciences

For a reference copy of the document with all sections, see nature.com/documents/nr-reporting-summary-flat.pdf

Life sciences study design

All studies must disclose on these points even when the disclosure is negative.

Sample size	Sample sizes were determined based on the previous similar studies in which the power was usually set at 80% and the level of significance was set at 5%. For exploratory experiments of B cell development or GC B cell response, usually 4-8 mice were sufficient to detect differences. For molecular biology experiment, typically 2-4 samples were sufficient.<br>Appropriate statistical methods used were described in the figure legends. The unpaired two-tailed student's t test was used for the difference analysis between two groups unless otherwise indicated, and the One-way ANOVA was used for the difference analysis of multiple groups. In ELISA experiments, the analysis of differences between the two groups at different time points was performed using two-way ANOVA.
Data exclusions	Occasionally, some mice that did not respond to humoral immunity well were excluded.
Replication	The number of replicates is given in the figure legends, ranging from 1 to 6 repeat experiments . All reported attempts at replication were successful.
Randomization	For experiments involving mice, mice were allocated into experimental groups according to the genotype of wild-type and knockout mice.<br>For experiments other than those involving mice, the samples were allocated into experimental groups as follows:<br>In RNA pull-down and EMSA assay, samples were allocated into experimental groups according to the added RNA sequences.<br>In RIP-qPCR assay, samples were allocated into experimental groups according to the used antibodies.<br>No randomization method was used.
Blinding	Blinding was used when immunizing mice. Data were collected and analyzed without using blinding to ensure that problems with samples could be addressed or alternate measurements for control and test samples.

Reporting for specific materials, systems and methods

We require information from authors about some types of materials, experimental systems and methods used in many studies. Here, indicate whether each material, system or method listed is relevant to your study. If you are not sure if a list item applies to your research, read the appropriate section before selecting a response.

Materials & experimental systems

n/a	Involved in the study
☐	☒ Antibodies
☐	☒ Eukaryotic cell lines
☒	☐ Palaeontology and archaeology
☐	☒ Animals and other organisms
☒	☐ Clinical data
☒	☐ Dual use research of concern

Methods

n/a	Involved in the study
☒	☐ ChIP-seq
☐	☒ Flow cytometry
☒	☐ MRI-based neuroimaging

Antibodies

Antibodies used

For B cell stimulation:
Purified NA/LE hamster anti-mouse CD40 (Clone: HM40-3, BD Pharmingen, Cat#553721, Lot: 2168842).

For western blot:
β-actin (1:2000 diluted, Proteintech, Cat#20536-1-AP, Lot: 00057922),
hnRNP F (1:1000 diluted, Thermo Fisher, Cat#PA522341, Lot: XK3754286C),
hnRNPA1 (1:1000 diluted, Clone: D21H11, Cell Signaling Technology, Cat#8443S, Lot: 2),
hnRNPA2B1 (1:1000 diluted, ABclonal, Cat#A1162, Lot: 0002840201),
p-IKKα/IKKβ (1:1000 diluted, Clone: C84E11, Cell Signaling Technology, Cat#2078S, Lot: 9),
IKKβ (1:1000 diluted, Clone: D30C6, Cell Signaling Technology, Cat#8943S, Lot: 4),
p-IKBα (1:1000 diluted, Clone: 14D4, Cell Signaling Technology, Cat#2859T, Lot: 18),
IKBα (1:1000 diluted, Cell Signaling Technology, Cat#9242S, Lot: 11).

For flow cytometry:
B220 APC-Cy7 (Clone: RA3-6B2, BD Pharmingen, Cat#562094, Lot: 9156958)
BP-1 PE (Clone: BP-1, BD Pharmingen, Cat#553735, Lot: 9277129)
CD43 FITC (Clone: eBioR2/60, eBioscience, Cat#11-0431-82, Lot: 2239975)
CD24 PerCP-Cy5.5 (Clone: M1/69, BD Pharmingen, Cat#562360, Lot: 1096358
IgD Biotin (Clone: 217-170, BD Pharmingen, Cat#553509, Lot: 5259651)
IgM PE-Cy7 (Clone: RMM-1, BioLegend, Cat#406514, Lot: B373773)
CD93 Biotin (Clone: AA4.1, eBioscience, Cat#13-5892-82, Lot: 2383260)
CD23 APC (Clone: B3B4, BioLegend, Cat#101620, Lot: B362281)
CD21/CD35 PerCP-Cy5.5 (Clone: 7G6, BD Pharmingen, Cat#562797, Lot: 9108719)
CD38 PerCP-Cy5.5 (Clone: 90/CD38, BD Pharmingen, Cat#562770, Lot: 9298312)
FAS-PE (Clone: 15A7, eBioscience, Cat#12-0951-83, Lot: 4280940)
CXCR4 APC (Clone: 2B11, eBioscience, Cat#17-9991-82, Lot: E12884-103)
CD83 FITC (Clone: Michel-17, eBioscience, Cat#11-0831-82, Lot: 4348501)
IgG1 FITC (Clone: A85-1, BD Pharmingen, Cat#553443, Lot: 6134528)
CD138 Biotin (Clone: 281-2, BD Pharmingen, Cat#553713, Lot: 5195797)
IgM Biotin (Clone: R6-60.2, BD Pharmingen, Cat#553406, Lot: 5104828)
Gr-1 Biotin (Clone: RB6-8C5, eBioscience, Cat#MA5-17969, Lot: E03075-1633)
CD3 Biotin (Clone: 17A2, BioLegend, Cat#100244, Lot: B198733)
IgD FITC (Clone: 11-26c, eBioscience, Cat#11-5993-82, Lot: 2452808)
GL-7 PE (Clone: GL-7, eBioscience, Cat#12-5902-82, Lot: 2305613)
CD40 FITC (Clone: 3/23, BD Pharmingen, Cat#553790, Lot: 6126533)
CD40 APC (Clone: 3/23, BioLegend, Cat#124612, Lot: B309981)
IgG3 FITC (Clone: R40-82, BD Pharmingen, Cat#553403, Lot: 7228736)
IgG1 APC (Clone: RMG1-1, BioLegend, Cat#406610, Lot: B203317)
GL-7 FITC (Clone: GL7, BD Pharmingen, Cat#553666, Lot: 5163878).

For ELISOT assay:
IgG1 Biotin (Cat#1144-08, Lot:E221-R111C)
IgM Biotin (Cat#1140-08, Lot: B1214-ZE28D)

For ELISA:
IgM-HRP (Cat#1021-05, Lot: I2015-YF97B)
IgG3-HRP (Cat#1101-05, Lot: B0512-WK86J)
IgG2b-HRP (Cat#1091-05, Lot: J1111-TI97F)
IgA-HRP (Cat#1040-05, Lot: C0919-PO51F)
IgG1-HRP (Cat#1071-05, Lot: B5312-VH57D)

Validation

All antibodies used for flow cytometry and western blot have validation statements on the manufacturer's website.
In our experiments, antibodies for western blot were validated by immunoblot of proteins according to protein ladder, and antibodies for flow cytometry were validated by flow cytometry analysis of mouse lymphocytes according to positive and negative populations.
Purified NA/LE hamster anti-mouse CD40 (HM40-3) used for B cell stimulation was validated by previous reports. For example:

1. Akiba H, Oshima H, Takeda K, et al. CD28-independent costimulation of T cells by OX40 ligand and CD70 on activated B cells. J Immunol. 1999; 162(12):7058-7066. (Clone-specific: (Co)-stimulation).
2. Ridge JP, Di Rosa F, Matzinger P. A conditioned dendritic cell can be a temporal bridge between a CD4+ T-helper and a T-killer cell. Nature. 1998; 393(6684):474-478. (Clone-specific: (Co)-stimulation).

Eukaryotic cell lines

Policy information about cell lines and Sex and Gender in Research

Cell line source(s)	3T3-L1 fibroblast cells were a gift from the Life Science Experimental Teaching Demonstration Center of Southern University of Science and Technology.<br>HEK293T cells were a gift from Yan Li's lab (Department of Biology, Southern University of Science and Technology).<br>3T3-L1 fibroblast cells (SCSP-5038) and HEK293T cells (SCSP-502) were purchased from the Cell Bank affiliated to Shanghai Institute of Biochemistry and Cell Biology.
Authentication	Cell lines were authenticated roughly by observation of morphology.
Mycoplasma contamination	The cell lines were not tested for mycoplasma contamination.
Commonly misidentified lines (See ICLAC register)	The study did not involved commonly misidentified lines.

Animals and other research organisms

Policy information about studies involving animals; ARRIVE guidelines recommended for reporting animal research, and Sex and Gender in Research

Laboratory animals	Cd19cre/+ (B6.129P2(C)-Cd19tm1(cre)Cgn/J) and Mb1cre/+ (B6.C(Cg)-Cd79atm1(cre)Reth/EhobJ) mice were obtained from Jackson Laboratories. Hnrnpf floxed mice were obtained from Nanjing Biomedical Research Institute of Nanjing University. To generate conditional knockout mice, the mice were crossed to Flp expressing mice to remove the neo-cassette and then crossed to Cd19cre/+ mice to delete exon 4 of Hnrnpf gene. Hnrnpa2b1 floxed mice were obtained from Cyagen and crossed with Mb1cre/+ to generate conditional knockout mice to delete its exons 2 to 6. Hnrnpa1 floxed mice were obtained from Gempharmatech and crossed with Mb1cre/+ to generate conditional knockout mice to delete its exons 2 to 11. Male and female mice of 6-12 weeks old were used for breeding and experiments. All mice were on a C57BL/6 background and housed in a pathogen-free, dark 12 hours/light 12 hours cycle, ambient temperature 20-26 ℃ and humidity ~70% environment in Laboratory Animal Center of the Southern University of Science and Technology. Male and female mice of 6-12 weeks old were used for breeding and experiments. For chimera assay, muMT- (B6.129S2-Ighmtm1Cgn/J) mice were obtained from Jackson Laboratories and recipients were wild-type C57BL/6J mice.
Wild animals	The study did not involved wild animals.
Reporting on sex	Both male and female mice were used in the experiments.
Field-collected samples	The study did not involve samples collected from the field.
Ethics oversight	All experimental animal operations were approved by the Institutional Animal Care and Use Committee of the Southern University of Science and Technology (ethics approval number: SUSTech-JY202205005).

Note that full information on the approval of the study protocol must also be provided in the manuscript.

Flow Cytometry

Plots

Confirm that:

☒ The axis labels state the marker and fluorochrome used (e.g. CD4-FITC).

☒ The axis scales are clearly visible. Include numbers along axes only for bottom left plot of group (a 'group' is an analysis of identical markers).

☒ All plots are contour plots with outliers or pseudocolor plots.

☒ A numerical value for number of cells or percentage (with statistics) is provided.

Methodology

Sample preparation	Single-cell suspensions were prepared from mouse spleens or bone marrow by removal of red blood cells using RBC lysis buffer (BioLegend) and passing through 70-µm nylon mesh.
Instrument	Beckman CytoFLEX S
Software	Data were collected using CytExpert software (version 2.3) and analyzed using FlowJo (version 10.8.1)

Cell population abundance	Purity of FACS-sorted follicelar B cells was analysed by flow cytometry.<br>Purity was >90%
Gating strategy	Supplementary Figure 1 shows exemplifying complete gating strategies of all analyzed B cells. Gates indicating boundaries between "positive" and "negative" are shown.

☒ Tick this box to confirm that a figure exemplifying the gating strategy is provided in the Supplementary Information.
